# Actinobacteria associated with Chinaberry tree are diverse and show antimicrobial activity

**DOI:** 10.1038/s41598-018-29442-2

**Published:** 2018-07-23

**Authors:** Ke Zhao, Jing Li, Meiling Shen, Qiang Chen, Maoke Liu, Xiaolin Ao, Decong Liao, Yunfu Gu, Kaiwei Xu, Menggen Ma, Xiumei Yu, Quanju Xiang, Ji Chen, Xiaoping Zhang, Petri Penttinen

**Affiliations:** 1 Department of Microbiology, College of Resource and Environmental Sciences, Sichuan Agricultural University, Yaan, 625000 P. R. China; 2 Biotechnology Center, Rice and Sorghum Research Institute, Sichuan Academy of Agricultural Sciences, Luzhou, 646100 P. R. China; 3 Zhejiang Provincial Key Laboratory of Carbon Cycling in Forest Ecosystems and Carbon Sequestration, School of Environmental & Resource Sciences, Zhejiang Agriculture & Forestry University, Linan, 311300 P. R. China; 4 Ecosystems and Environment Research Programme, University of Helsinki, Helsinki, Fin-00014 Finland

## Abstract

Many actinobacteria produce secondary metabolites that include antimicrobial compounds. Since most of the actinobacteria cannot be cultivated, their antimicrobial potential awaits to be revealed. We hypothesized that the actinobacterial endophyte communities inside *Melia toosendan* (Chinaberry) tree are diverse, include strains with antimicrobial activity, and that antimicrobial activity can be detected using a cultivation independent approach and co-occurrence analysis. We isolated and identified actinobacteria from Chinaberry, tested their antimicrobial activities, and characterized the communities using amplicon sequencing and denaturing gradient gel electrophoresis as cultivation independent methods. Most of the isolates were identified as *Streptomyces* spp., whereas based on amplicon sequencing the most abundant OTU was assigned to *Rhodococcus*, and *Tomitella* was the most diverse genus. Out of the 135 isolates, 113 inhibited the growth of at least one indicator organism. Six out of the 7577 operational taxonomic units (OTUs) matched 46 cultivated isolates. Only three OTUs, *Streptomyces* OTU4, OTU11, and OTU26, and their corresponding isolate groups were available for comparing co-occurrences and antimicrobial activity. *Streptomyces* OTU4 correlated negatively with a high number of OTUs, and the isolates corresponding to *Streptomyces* OTU4 had high antimicrobial activity. However, for the other two OTUs and their corresponding isolate groups there was no clear relation between the numbers of negative correlations and antimicrobial activity. Thus, the applicability of co-occurrence analysis in detecting antimicrobially active actinobacteria could not be proven.

## Introduction

Free-living and endophytic actinobacteria are common in terrestrial and marine environments. Most of the actinobacteria either cannot be cultivated with current methods or are overgrown by bacteria that grow faster if cultivation conditions are not specifically chosen. In culture-dependent studies most of the isolates have been identified as members of the genus *Streptomyces*. Culture-independent methods, for example amplicon sequencing targeting the bacterial 16S rRNA gene, have shown that *Streptomyces* species form only a small fraction of actinobacterial communities in soil, marine sediments, and inside a plant^1,2^, and that revealing the diversity of actinobacteria necessitates a focused approach. When the 16S rRNA gene was amplified using actinobacteria specific primers for amplicon sequencing, rice plant (*Oryza sativa* L.) was noticed to host almost four hundred actinobacterial operational taxonomic units (OTUs) at 97% similarity^3^, i.e. at the similarity level commonly applied in bacterial species delineation.

Due to the increase in antibiotic resistance among pathogens, discovering novel antimicrobials is desirable^4^. Many actinobacteria produce secondary metabolites that include antimicrobial compounds. However, since most of the actinobacteria cannot be cultivated, their antimicrobial potential awaits to be revealed. To find strains that produce novel compounds or could be used in biocontrol of plant diseases, medicinal plants with antimicrobial properties are of particular interest, since the endophytes of the plants may be involved in producing the antimicrobial compounds^5–7^, and the environment inside the plant is potentially selective. Although antimicrobial compound production is common among actinobacteria, it has been estimated that novel compounds are produced in less than one in 10^7^ fermentation broths of randomly selected actinobacteria^8^. To lessen the needed cultivation effort, methods to target antimicrobially active actinobacteria are called for.

Molecular methods based on the detection of genes that are needed to produce antimicrobially active compounds might be useful in detecting strains with antimicrobial activity. However, clear correlation between antimicrobial activity and the presence of these genes has not been found. The members of microbial community interact with each other and the environment, and characterizing the interactions in the community may reveal new insights into the activities of its members. Microbes may interact via volatile compounds, yet the activity of soluble compounds is usually stronger^9^. Moisture increases motility, creating more possibilities for interactions. Plants with their microbial endophytes offer a possibility to study within community interactions in a limited space with sufficient moisture. Furthermore, actinobacterial endophyte communities that include producers of antimicrobial compounds are expected to display social and asocial interactions^10^.

We hypothesized that the actinobacterial endophyte communities inside traditional medicinal plant *Melia toosendan* (Chinaberry) are diverse, and include strains with antimicrobial activity. We expected that antimicrobial activity can be detected using a cultivation independent approach and co-occurrence analysis: asocial interaction, where one strain inhibits the growth of another, will result in negative correlation between the strains. To study this, we (i) isolated strains from Chinaberry, identified the strains using 16S rRNA gene sequencing, and tested their antimicrobial activities, and (ii) applied denaturing gradient gel electrophoresis and amplicon sequencing targeting the 16S rRNA gene as cultivation independent approaches. The sequences from amplicon sequencing were clustered into OTUs, and representative sequences were compared with sequences from the isolated strains. Co-occurrences of the OTUs were analyzed, and the results from co-occurrence and antimicrobial activity analyses were compared.

## Materials and Methods

### Sample collection and DNA extraction

Root, stem, leaf, fruit, and bark samples were cut from healthy *Melia toosendan* Sieb.et Zucc trees at seven sites in Sichuan, China (Table S1). To ensure the endophytic nature of the isolates, the cut ends were sealed with wax. Samples were placed in sterile bags, brought into laboratory on ice and surface sterilized within 24 as previously described^11^.

20 g of fresh surface-sterilized plant tissue was ground into fine powder in liquid nitrogen and hydrolyzed as previously described^12,13^ except that root, bark and stem samples were incubated for 9 h, and leaf and fruit samples for 7 h in dark at 28 °C. DNA was extracted from the hydrolyzed samples using DNeasy Plant Mini Kit (QIAGEN, Germany) according to manufacturer’s instructions.

### Denaturing gradient gel electrophoresis

Nested PCR for denaturing gradient gel electrophoresis (DGGE) was done as described with some modifications^14^. The first amplification was carried out with primers F243 (5′-GGATGAGCCCGCGGCCTA-3′) and R1186 (5′-CTTCCTCCGAGTTGACCC-3′)^15,16^. PCR mixture consisted of 1× buffer (1.0 mM Mg^2+^), dNTP mixture (200 μM each deoxynucleoside triphosphate), 0.2 μM each primer, 30 μg mL^−1^ bovine serum albumin, 1 U of Taq DNA polymerase (TaKaRa, China), and 0.5 μL template DNA in a final volume of 20 μL. Initial denaturation at 95 °C for 5 min was followed by 20 cycles of denaturation at 95 °C for 1 min, annealing at 65 °C for 1 min, and extension at 72 °C for 2 min, followed by 20 cycles during which the annealing temperature was lowered from 65 to 55 °C with 0.5 °C each cycle, 15 cycles with annealing at 55 °C for 45 s, and a final extension at 72 °C for 10 min. The second amplification was carried out as the first one except with primers F907 (AAACTCAAAGGAATTGACGG) with a GC-clamp and R1186 (CTTCCTCCGAGTTGACCC)^16,17^, using 1 μl of the amplification mixture from first PCR as template. Amplification of fragments with expected size was verified by electrophoresis in 1% agarose gel.

PCR products were separated in an 8% (w/v) polyacrylamide gel with a 30–60% denaturant gradient in Tris acetate EDTA (TAE) buffer for 8 h at 60 °C and 160 V using a Dcode Universal Mutation Detection System (Bio-Rad Laboratories, USA). After electrophoresis, the gels were silver stained as described previously^18^. Gel images were acquired using a Gel Doc imaging system (Bio-Rad Laboratories, USA). DGGE bands were excised and reamplified using the 907f and 1186r primers without the GC-clamp. PCR products were purified with a Gel Purification Kit (TaKaRa, China), and cloned into pMD19-T vectors (TaKaRa, China) according to the manufacturer’s instructions. The vectors were transformed into *Escherichia coli* DH5a competent cells, clones were picked, and the presence of correct size inserts was verified by PCR using the primers M13F and M13R. Clones were sequenced at Suzhou GENEWIZ Biological Technology Co., Ltd. (Suzhou, China). Clone sequences were compared with sequences in the National Center for Biotechnology Information (NCBI) database using BLASTN.

### Amplicon sequencing and sequence data analysis

Actinobacteria-specific primers Com2xF and Ac1186R with Illumina adapters were used to amplify an approximately 270 bp fragment of 16S rRNA gene^16,17^. PCR mixture consisted of 1× buffer (1.5 mM Mg^2+^), dNTP mixture (250 μM each deoxynucleoside triphosphate), 0.5 μM each primer, 60 μg mL^−1^ bovine serum albumin, 2 U of Taq DNA polymerase (TaKaRa, China), and 1 μL template DNA in a final volume of 50 μL. The amplification was as follows: 5 min at 95 °C; 30 cycles of 1 min at 92 °C, 1 min at 52 °C, 1 min at 72 °C; 72 °C for 15 min and cooling at 10 °C. Illumina Miseq paired end 2*250 sequencing was done at GENEWIZ (Suzhou, China).

The sequence data was analyzed at IT Center for Science (CSC), Finland, using Mothur v.1.36.1^19^ standard operating protocol^20^, except that reads were trimmed using the sequences AAACTCAAAGGAATTGACGG^17^ and CTTCCTCCG AGTTGACCC^16^ in the forward and reverse primers, respectively, after making the contigs. Sequences were aligned to SILVA v119 reference file^21,22^. Chimeras were removed using the UCHIME algorithm^23^.

Sequences were classified according to SILVA v119 reference file. Taxa that were not targeted in sequencing were removed. First, chloroplast, mitochondria, unknown, Archaea and Eukaryota were removed. After examining the taxonomic assignments of the remaining sequences, non-actinobacterial taxa (*Armatimonadetes*, *Bacteroidetes*, *Chlamydiae*, *Chlorobi*, *Cyanobacteria*, *Firmicutes*, *Planctomycetes*, *Proteobacteria*, *Verrucomicrobia*) were removed. Sequences were clustered into operational taxonomic units (OTUs) at 97% sequence similarity level. After removing singleton OTUs (OTUs with only one sequence) the number of OTUs was 7577. After subsampling so that all the samples had the same number of sequences as the sample with smallest number of sequences (Xindou root sample, 65542 sequences) the number of OTUs was 2671.

To estimate alpha diversity, Chao1 richness estimator and the inverse Simpson diversity index were generated using Mothur v.1.36.1. Sample coverages were estimated by rarefaction. Differences in numbers of OTUs, numbers of sequences and diversity indices between samples from different plant organs (bark, fruit, leaf, root, stem) were tested with ANOVA in R version 3.1.3^24^.

Differences between actinobacterial OTU communities in different plant organs were tested using PRIMER v.7 software^25^ with the add-on package PERMANOVA+^26^. Sub-sampled OTU data were log(x + 1) -transformed before calculating the Bray-Curtis dissimilarities between the samples.

Numbers of OTUs in different organs were visualized with a Venn diagram generated in R version 3.1.3 using the package VennDiagram^27^. Taxonomic compositions of the OTU communities at order level were visualized in R, and tested with Kruskal-Wallis test and Nemenyi post hoc test using the package PMCMR^28^ in R. OTUs with abundance greater than 0.001% of the total abundance were selected for further analysis. The taxonomic relations and abundances of OTUs were visualized using Cytoscape v. 3.3.0^29^.

### Isolation and identification of endophytic actinobacteria

Plant samples were surface sterilized as previously described^11^. Aliquots from the final rinse were inoculated on ISP_2_ media and incubated at 28 °C for 3-4 weeks. The surface sterilization was considered successful if there was no growth on the media. Samples were aseptically crumbled into smaller pieces using commercial blender. Subsequently, the tissue fragments were placed on ten selective isolation media: CMC-sodium pyruvate agar (CMC-Na: 2.5 g; sodium pyruvate 2.0 g; KNO_3_ 0.25 g; K_2_HPO_4_ 0.6 g; MgSO_4_ 0.2 g; KH_2_PO_4_ 0.2 g; CaCl_2_ 0.5 g; FeSO_4_ *7 H_2_O 0.01 g; proline 1 g; ddH_2_O 1 L; pH 7.2; this study), dulcite-proline agar (Dulcite 2.0 g; Proline 0.5 g; K_2_HPO_4_ 0.3 g; NaCl 0.3 g; MgSO_4_ 1 g; CaCl_2_ 1 g; FeCl_3_ 0.01 g; ddH_2_O 1 L; pH 7.2; this study), xylose-arginine agar^30^, histidine-raffinose agar (Qin *et al*.^31^), modified Gause I agar^31^, tap water yeast extract agar (TWYE)^32^, alanine-arginine agar^30^, sodium citrate agar^33^, sodium succinate agar^33^ and chitin agar^34^. All isolation media were supplemented with nalidixic acid and K_2_Cr_2_O_7_ (50 µg ml^−1^) to inhibit the growth of non-actinobacteria. The isolation plates were incubated at 28 °C for 3-4 weeks. Based on the morphological features, odor and pigment, actinobacterial colonies were selected and purified. The pure cultures were stored on ISP_4_ slope medium at 4 °C.

The isolates were dereplicated by cultural and morphological characteristics, including morphology and color of aerial mycelium, characteristics of colonies on the plate, spore mass color, color of diffusible pigments, and sporophore and spore chain morphology according to Bergey’s manual of determinative bacteriology^35^. Visual observation was done using light microscopy (Olympus CX31, Olympus Corp., Japan), and the morphological features of spores and mycelia were examined by scanning electron microscope (Zeiss EVO 40 EP, Carl Zeiss AG, Germany)^36^ after incubation on ISP_4_ medium at 28 °C for 10 days. Representative isolates were identified by 16S rRNA gene sequencing as described earlier^14^.

### Evaluation of antimicrobial activity

Representative isolates were tested for their antimicrobial activity against nine indicator organisms: *Colletotrichum orbiculare* (SAUM 5312), *Fusarium oxysporum* (SAUM 5429), *Altemaria solani* (SAUM 5230), *Magnaporthe grisea* (SAUM 5411), *Curvularia lunata* (SAUM 5429), *Gibberella saubinetii* (SAUM 5456), *Bacillus subtilis* (SAUM 5139), *Staphylococcus aureus* (ATCC25923) and *Escherichia coli* (ATCC 35218). The antimicrobial activities were tested as described earlier^37,38^. All strains were tested in triplicates.

### Detection and analysis of biosynthetic gene fragments

Primers K1F (5′-GTSCCSGTSCCRTGSSCYTCSAC-3′) and M6R (5′-GCSATGG AYCCSCARCARCGSVT-3′)^39^, KSaF (5′-TSGCSTGCTTGG AYGCSATC-3′) and KSaR (5′-TSGCSTGCTTG GAYGCSATC-3′)^40^, A3F (5′-GCSTACSYSATSTACACSTCSGG-3′) and A7R (5′-SASGTC VCCSGTSCGCTAS-3′)^41^, and B4F (5′-TTCCCS CGSTACCASATCGGSGAG-3′) and B7R (5′-GSGGGATSWMCCAGWACCASC C-3′)^42^ were employed to amplify the genes for the KS domains of type I polyketide synthase gene (PKS I), the KS domains of PKS II, the adenylation domains of non-ribosomal peptide synthetase gene (NRPS), and FADH_2_-dependent halogenase gene of halogenation pathway. PCR amplification was done in 20 µl PCR reaction mixture consisting of 1 × buffer with 1.5 mM Mg^2+^, 0.5 mM dNTP mixture, 1 µM of primers, 1 µl of template DNA, 1 U Taq polymerase and 1.25 µg µl^−1^ BSA. The reaction conditions for the PCR were as follows: (1) PKS I: 5 min at 95 °C, followed by 35 cycles of 30 s at 95 °C, 2 min at 55 °C and 4 min at 72 °C, followed by 10 min at 72 °C; (2) PKS II: 2 min at 96 °C, followed by 30 cycles of 1 min at 96 °C, 2 min at 60 °C, 90 s at 73 °C, followed by 10 min at 72 °C; (3) NRPS: 5 min at 95 °C, followed by 30 cycles of 1 min at 95 °C, 90 s at 59 °C, 2 min at 72 °C, followed by 10 min at 72 °C; (4) Halogenase: 5 min at 95 °C, followed by 35 cycles of 1 min at 95 °C, 1 min at 60 °C, 2 min at 72 °C, followed by 10 min at 72 °C.

Bray–Curtis dissimilarities of the isolates that inhibited the growth of at least one indicator strain were calculated based on the presence/absence of inhibition using the R-package vegan^24,43^. Grouping of the isolates carrying different combinations of halogenase, PKSI, PKSII and NPRS genes were visualized with non-metric multidimensional scaling.

### Comparison of OTUs and isolates

The 16S rRNA gene sequences of database entries that contained a region 100% similar to the 16S rRNA gene sequences of cultivated isolates were compiled and formatted as a BLAST database using NCBI BLAST version 2.4.0 at CSC, after which the representative sequences of OTUs from 16S rRNA gene amplicon sequencing were compared with the database sequences using BLASTN.

Differences in the numbers of positive antimicrobial activity tests of isolate groups corresponding to OTU4, OTU11 and OTU26 were tested with Likelihood Ratio chi-square test first for all groups and then as partitions (OTU4-like isolates to OTU26-like, summed OTU4-like and OTU26-like to OTU11-like) to estimate the significance of between group differences.

OTUs that were detected in more than half of the samples were selected for a co-occurrence analysis. Spearman correlations between all OTU pairs were calculated and p-values were adjusted for multiple comparisons using Benjamini-Hochberg correction with the R package psych^44^. Differences in the numbers of negative and positive correlations of OTU4, OTU11 and OTU26 were tested with Fisher’s exact test first with all three OTUs and then pairwise. For the pairwise comparisons, statistical significance was taken significant as p < 0.05/3.

### Data availability

The 16S rRNA gene sequences from DGGE analysis were deposited in NCBI Nucleotide database under the accession numbers KY388868-KY388922 (www.ncbi.nlm.nih.gov). The amplicon sequencing data were deposited in European Nucleotide Archive in the study PRJEB21654 (www.ebi.ac.uk/ena). The 16S rRNA gene sequences of the isolates were deposited in NCBI under the accession numbers MF624486 - MF624620. The sequences of halogenase, PKSI, PKSII and NPRS genes were deposited in NCBI under the accession numbers MF802345 - MF802484.

## Results

To characterize actinobacterial community inside the medicinal plant *Melia toosendan*, we applied both culture-dependent and culture-independent methods. Actinobacteria were identified by amplicon sequencing, denaturing gradient gel electrophoresis (DGGE) and Sanger sequencing, all targeting the 16S rRNA gene using actinobacteria specific primers. Sequences from amplicon sequencing were grouped to 7577 operational taxonomic units (OTUs) at 97% similarity level. The average number of actinobacterial OTUs in different *M. toosendan* organs ranged from 177 to 325 (Table S2). Variation in the number of OTUs was high, and differences between organs were not statistically significant. Based on rarefaction, the average sample coverage was 99.95% ± 0.03%, indicating that the results represented well the actinobacterial communities of *M. toosendan*. With the exception of bark, the diversity of actinobacterial communities in different organs was approximately on the same level. However, as the variation was highest in the bark samples, the difference between bark and other organs was not statistically significant. According to the DGGE analysis, the Shannon diversity of the actinobacterial communities in different organs ranged from 2.46 to 3.36 with no statistically significant differences between the organs.

**Figure 1:**
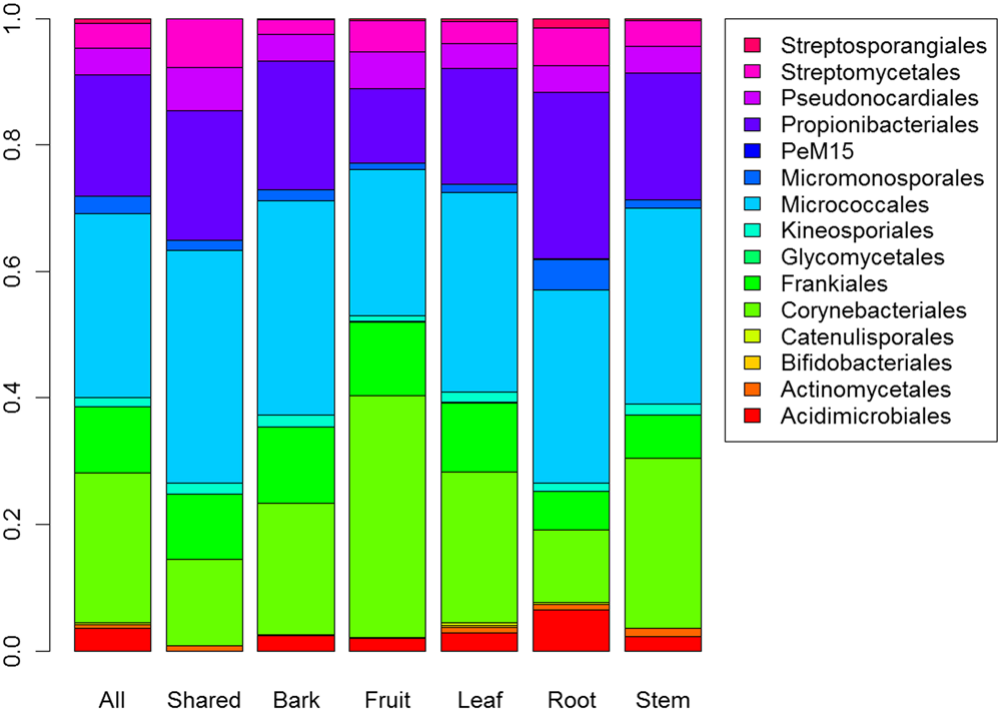
The taxonomic composition at order level of the actinobacterial OTU communities inside *Melia toosendan* tree detected by targeted amplicon sequencing of 16S rRNA gene. All = all OTUs; Shared = OTUs detected in every plant organ; Bark, Fruit, Leaf, Root, Stem = OTUs detected in individual plant organs.

To characterize the actinobacterial communities in plant organs, the amplicon sequencing data was subsampled to even sequencing depth and log + 1 transformed for statistical analyses, resulting in 2671 OTUs. In the subsampled data, the number of OTUs was 1262 in the bark, 649 in the fruit, 702 in the leaf, 1052 in the root, and 638 in the stem. Altogether 117 OTUs, all representing the class *Actinobacteria*, were detected in every organ (Fig. S1). Only the most abundant OTU, *Rhodococcus* sp. OTU1, was detected in every sample. The percentage of OTUs detected in one organ only was 54% in the bark, 39% in the fruit, 32% in the leaf, 45% in the root, and 24% in the stem. Almost all of these were low abundance OTUs that were detected in one or two samples only, evidencing the large variation. Permutational ANOVA did not reveal any statistically significant differences in community composition (p = 0.0617). However, at order level the taxonomic compositions of the OTU communities detected in *M. toosendan* bark, fruit, leaf, root, and stem were different (p < 0.05) (Fig. 1). Pairwise comparisons revealed that the fruit and root communities were different from the other communities (Table 1).

**Table 1:** P-values from the pairwise Nemenyi tests of the taxonomic composition at order level of the actinobacterial OTU communities in *Melia toosendan* bark, fruit, leaf, root and stem.

	Bark	Fruit	Leaf	Root
Fruit	**<0.01**			
Leaf	0.62	**<0.05**		
Root	**<0.01**	**<0.01**	**<0.01**	
Stem	0.96	**<0.01**	0.98	**<0.01**

Statistically significant differences are in bold.

**Figure 2:**
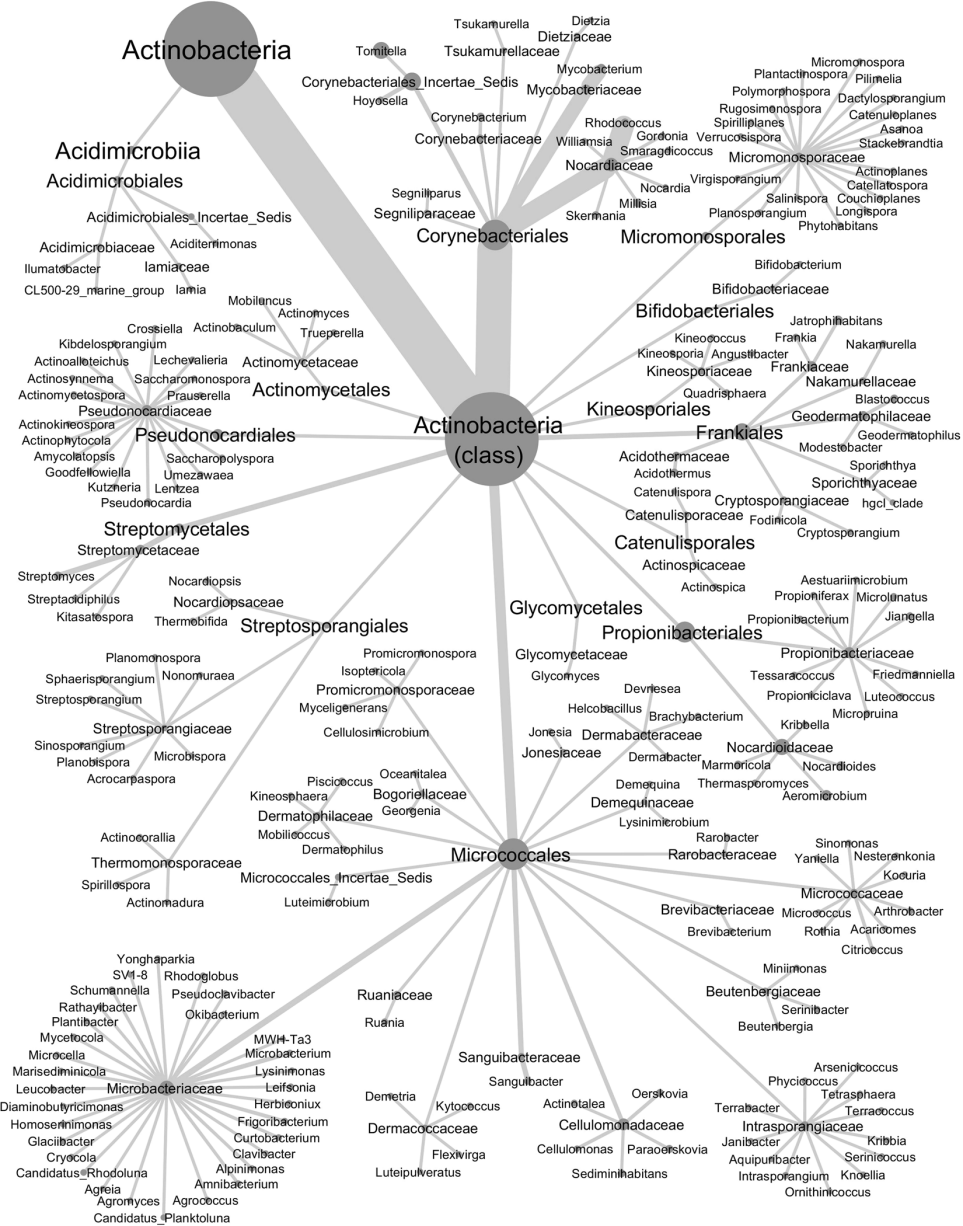
Taxonomic assignments of actinobacterial OTUs from *Melia toosendan* based on OTUs with abundance greater than 0.001% of the total abundance. Node size is proportional to the number of OTUs assigned to the node. Edge width is proportional to the abundance of OTUs.

As expected, the genus composition of the isolate group, determined by 16S rRNA gene sequencing, was different from those detected by amplicon sequencing and DGGE. Out of the 135 isolates, 93, 21 and one represented thirty *Streptomyces*, eight *Micromonospora* and *Rhodococcus enclensis* species, respectively (Fig. S3, Table S3). The rest nineteen isolates represented fifteen species in thirteen genera. In the amplicon sequencing data, *Corynebacteriales* and *Micrococcales* were equally diverse with 2062 and 2152 OTUs, respectively, yet *Corynebacteriales* were approximately six times more abundant than *Micrococcales* (Fig. 2). At genus level, *Tomitella* with 899 OTUs was over two times more diverse and over four times more abundant than *Aeromicrobium*, the second most diverse genus. Out of the 7577 OTUs, 195, four and 139 were members of the genera *Streptomyces*, *Micromonospora* and *Rhodococcus*, respectively (Table S4). Out of the 56 sequenced DGGE bands fourteen were affiliated with *Streptomyces*, one with *Micromonospora*, six with *Rhodococcus*, and the rest represented sixteen genera (Fig. S3).

The representative sequences of six OTUs were 100% similar to the database entries that had sequence regions 100% similar to the cultivated isolates (Table 2). Through this indirect comparison, in total the six OTUs matched 46 cultivated isolates. Since sequences similar to the relatively short 16S rRNA gene amplicons are found on different actinobacterial species, the six OTUs may have represented more than six species. For example, OTU4 matched both *Streptomyces geysiriensis* and *S. enissocaesilis*. Although the average abundances of *Streptomyces* OTU4, OTU11 and OTU26 were low in the root, stem and root samples, respectively, strains similar to them were isolated from all the organs. Strains similar to *Cellulosimicrobium* OTU134 were isolated from samples in which OTU134 was not detected, whereas a strain similar to *Arthrobacter* OTU205 was isolated from a sample where the abundance of OTU205 was at its highest.

**Table 2:** OTUs 100% similar to database entries that had sequence regions 100% similar to the actinobacteria isolated from the bark, fruit, leaf, root and stem of *Melia toosendan*.

OTU	Site^a^	Organ^b^	Isolate	Site	Organ	Assignment
Otu4	2–6	1–5	SCAU8224	Ya’an	Bark	*Streptomyces enissocaesilis* NRRL B-16365^T^ (DQ026641)
			SCAU8184	Suining	Root	*Streptomyces enissocaesilis* NRRL B-16365^T^ (DQ026641)
			SCAU8187	Suining	Bark	*Streptomyces enissocaesilis* NRRL B-16365^T^ (DQ026641)
			SCAU8188	Suining	Leaf	*Streptomyces enissocaesilis* NRRL B-16365^T^ (DQ026641)
			SCAU8174	Suining	Fruit	*Streptomyces enissocaesilis* NRRL B-16365^T^ (DQ026641)
			SCAU8175	Jingtang	Stem	*Streptomyces enissocaesilis* NRRL B-16365^T^ (DQ026641)
			SCAU8191	Jingtang	Root	*Streptomyces enissocaesilis* NRRL B-16365^T^ (DQ026641)
			SCAU8176	Xindou	Bark	*Streptomyces enissocaesilis* NRRL B-16365^T^ (DQ026641)
			SCAU8192	Xindou	Stem	*Streptomyces geysiriensis* NBRC 15413^T^ (AB184661)
			SCAU8193	Xindou	Leaf	*Streptomyces enissocaesilis* NRRL B-16365^T^ (DQ026641)
			SCAU8195	Xichang	Root	*Streptomyces enissocaesilis* NRRL B-16365^T^ (DQ026641)
			SCAU8196	Mianyang	Leaf	*Streptomyces enissocaesilis* NRRL B-16365^T^ (DQ026641)
			SCAU8198	Ya’an	Stem	*Streptomyces enissocaesilis* NRRL B-16365^T^ (DQ026641)
			SCAU8199	Suining	Stem	*Streptomyces enissocaesilis* NRRL B-16365^T^ (DQ026641)
			SCAU8202	Suining	Stem	*Streptomyces enissocaesilis* NRRL B-16365^T^ (DQ026641)
			SCAU8177	Jingtang	Leaf	*Streptomyces enissocaesilis* NRRL B-16365^T^ (DQ026641)
			SCAU8178	Mianyang	Fruit	*Streptomyces enissocaesilis* NRRL B-16365^T^ (DQ026641)
			SCAU8206	Mianyang	Root	*Streptomyces geysiriensis* NBRC 15413^T^ (AB184661)
			SCAU8211	Jingtang	Bark	*Streptomyces enissocaesilis* NRRL B-16365^T^ (DQ026641)
			SCAU8215	Suining	Root	*Streptomyces enissocaesilis* NRRL B-16365^T^ (DQ026641)
			SCAU8320	Mianyang	Stem	*Streptomyces enissocaesilis* NRRL B-16365^T^ (DQ026641)
			SCAU8221	Ya’an	Fruit	*Streptomyces enissocaesilis* NRRL B-16365^T^ (DQ026641)
Otu11	1–7	1–5	SCAU8119	Ziyang	Leaf	*Streptomyces anulatus* NRRL B-2000^T^ (DQ026637)
			SCAU8137	Xindou	Leaf	*Streptomyces anulatus* NRRL B-2000^T^ (DQ026637)
			SCAU8134	Jingtang	Bark	*Streptomyces flavovirens* NBRC 3716^T^ (AB184834)
			SCAU8148	Jingtang	Bark	*Streptomyces flavovirens* NBRC 3716^T^ (AB184834)
			SCAU8135	Ya’an	Fruit	*Streptomyces flavovirens* NBRC 3716^T^ (AB184834)
			SCAU8222	Xindou	Stem	*Streptomyces anulatus* NRRL B-2000^T^ (DQ026637)
			SCAU8186	Suining	Root	*Streptomyces fulvissimus* NBRC 3716 ^T^ (AB184834)
			SCAU8234	Jingtang	Leaf	*Streptomyces anulatus* NRRL B-2000^T^ (DQ026637)
			SCAU8235	Ziyang	Root	*Streptomyces anulatus* NRRL B-2000^T^ (DQ026637)
Otu26	1–7	1–5	SCAU8108	Suining	Stem	*Streptomyces hydrogenans* NBRC 13475^T^ (AB184868)
			SCAU8129	Ya’an	Leaf	*Streptomyces hydrogenans* NBRC 13475^T^ (AB184868)
			SCAU8203	Ya’an	Leaf	*Streptomyces hydrogenans* NBRC 13475^T^ (AB184868)
			SCAU8159	Xindou	Bark	*Streptomyces hydrogenans* NBRC 13475^T^ (AB184868)
			SCAU8157	Xichang	Root	*Streptomyces hydrogenans* NBRC 13475^T^ (AB184868)
			SCAU8130	Mianyang	Fruit	*Streptomyces hydrogenans* NBRC 13475^T^ (AB184868)
			SCAU8161	Jingtang	Root	*Streptomyces hydrogenans* NBRC 13475^T^ (AB184868)
			SCAU8207	Ziyang	Root	*Streptomyces hydrogenans* NBRC 13475^T^ (AB184868)
			SCAU8180	Ziyang	Bark	*Streptomyces hydrogenans* NBRC 13475^T^ (AB184868)
			SCAU8182	Jingtang	Stem	*Streptomyces hy drogenans* NBRC 13475^T^ (AB184868)
Otu134	1–5	1–5	SCAU8116	Jingtang	Fruit	*Cellulosimicrobium funkei* ATCC BAA-886^T^ (AY501364)
			SCAU8208	Xindou	Bark	*Cellulosimicrobium funkei* ATCC BAA-886^T^ (AY501364)
Otu205	1, 3–5	1–5	SCAU8248	Suining	Bark	*Arthrobacter pascens* ATCC 13346^T^ (X80740)
Otu256	1–3, 6	2–4	SCAU8105	Xindou	Leaf	*Nocardiopsis alba* DSM 43377^T^ (X97883)
			SCAU8165	Jingtang	Leaf	*Nocardiopsis alba* DSM 43377^T^ (X97883)

^a^1 = Jingtang, 2 = Mianyang, 3 = Suining, 4 = Xichang, 5 = Xindou, 6 = Ya’an, 7 = Ziyang; ^b^1 = Bark, 2 = Fruit, 3 = Leaf, 4 = Root, 5 = Stem.

**Figure 3:**
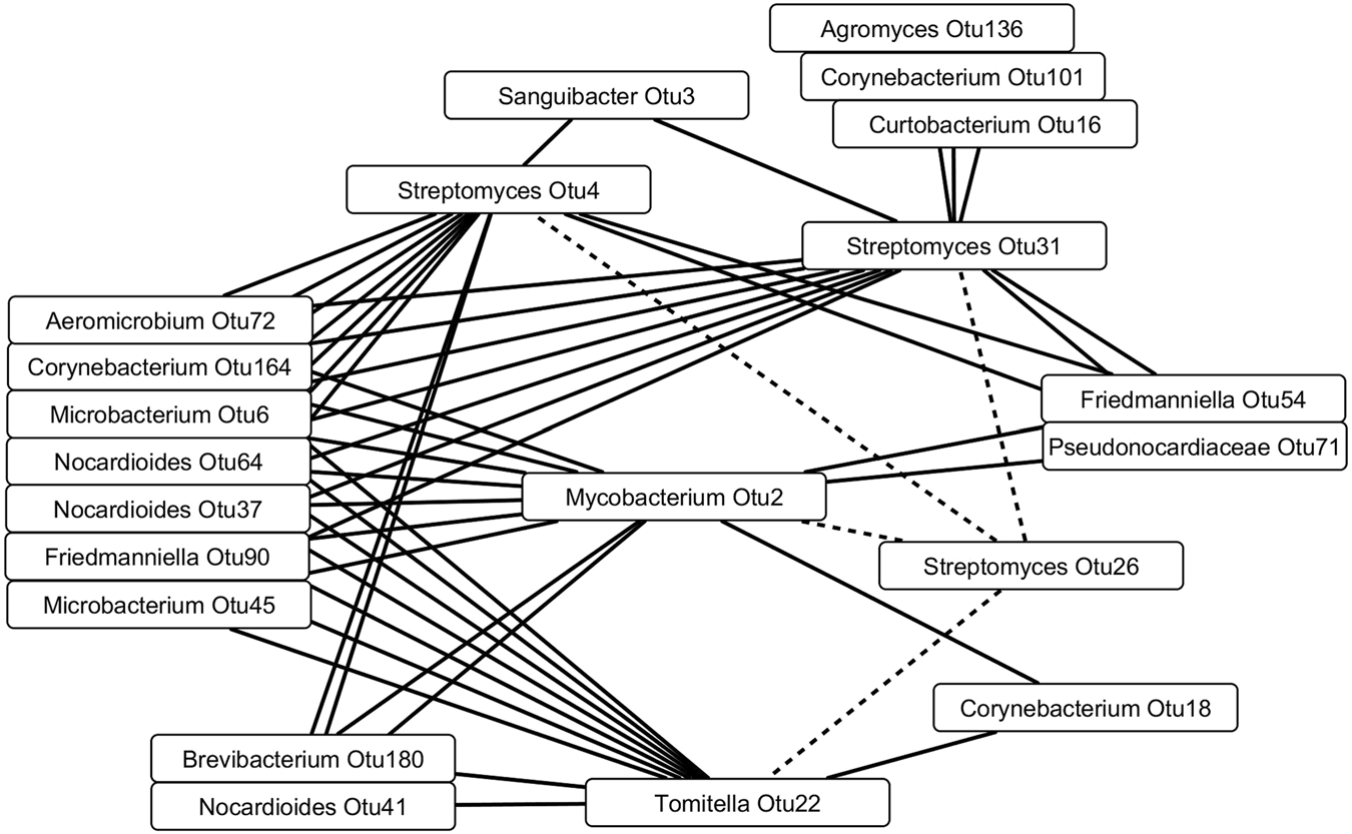
Spearman correlations of *Mycobacterium* OTU2, *Streptomyces* OTU4 and OTU31 and *Tomitella* OTU22 from *Melia toosendan* actinobacterial community. Solid and dashed lines indicate negative and positive correlations, respectively.

To characterize the OTU community further, 39 OTUs that were detected in more than half of the samples were selected for a co-occurrence analysis. Altogether 191 positive and 51 negative statistically significant Spearman correlations were detected, ranging from two to 23 correlations per OTU. Four OTUs (*Mycobacterium* OTU2, *Streptomyces* OTU4 and OTU31, *Tomitella* OTU22) correlated negatively with ten to thirteen OTUs and positively with *Streptomyces* OTU26 (Fig. 3). The other 35 OTUs correlated mostly or only positively.

To estimate the antimicrobial potential of the *M. toosendan* endophytes, the growth inhibiting activity of the isolates was tested (Table S5). The percentage of growth inhibiting isolates ranged from 19% against *Escherichia coli* to 54% against *Curvularia lunata*. Three *Streptomyces* isolates, *S. enissocaesilis* SCAU8202 and SCAU8176, and *S. costaricanus* SCAU8216 inhibited the growth of all nine indicator organisms (Table S5). A group of 24 isolates, including twelve *Streptomyces* and twelve rare actinobacteria isolates, did not inhibit any. The average inhibition zone width of fifteen isolates, including one *Micromonospora* isolate, two *Pseudonocardia* isolates and twelve *Streptomyces* isolates, was over ten mm (Table S5). Based on the width of the inhibition zone, the antimicrobial activity of *S. costaricanus* SCAU8216 was highest.

The comparison of co-occurrence analysis and antimicrobial activity results was done using three OTUs and their corresponding isolates; the other OTUs that corresponded to isolates were not included in the co-occurrence analysis since they were not detected in over half of the samples. The 22 isolates corresponding to OTU4 inhibited from five to nine indicator organisms, the ten OTU26 corresponding isolates inhibited from none to seven, and the nine OTU11 corresponding isolates from none to five. The differences in the numbers of positive antimicrobial activity tests between the isolate groups corresponding to *Streptomyces* OTU4, OTU11, and OTU26 were statistically significant (Table 3). The number of negative correlations of *Streptomyces* OTU4 was greater than those of *Streptomyces* OTU11 and OTU26 (p < 0.05).

**Table 3:** The numbers of negative correlations and similar isolates of *Streptomyces* OTU4, OTU11 and OTU26, and the numbers of positive antimicrobial activity (AMA) tests by the isolates. Different uppercase letters in a column indicate statistically significant difference (p < 0.05).

OTU	Negative correlations	Similar isolates	Positive AMA tests
OTU4	12^a^	22	164^a^
OTU11	0^b^	9	21^c^
OTU26	3^b^	10	45^b^

The presence of four genes linked to the production of antimicrobial compounds was assessed. PKSII was detected in 60%, PKSI in 25%, and NPRS and halogenase in 15% of the isolates (Table S3). None of the isolates carried all four genes. The isolates *Micromonospora schwarzwaldensis* SCAU8147, *Streptomyces enissocaesilis* SCAU8193 and SCAU8195, *S. roseolus* SCAU8117, and *S. tendae* SCAU8101 carried three of the genes. Thirty-three isolates, including fifteen *Streptomyces* and eighteen rare actinobacteria isolates, did not carry any of the genes. The isolates carrying different combinations of the genes did not cluster together in the non-metric multidimensional scaling that visualized the Bray-Curtis dissimilarities based on the presence-absence of growth inhibiting activities (Fig. S4).

## Discussion

We characterized the actinobacterial endophytes of the traditional medicinal plant *Melia toosendan*, estimated their antimicrobial activity, and related the results from cultivation dependent and independent approaches. The actinobacteria community inside *Melia toosendan* was approximately equally diverse in bark, fruit, leaf, root, and stem. Even though almost half of the operational taxonomic units (OTUs) in each plant organ was detected in that organ only, due to the large variation the OTU communities could not be considered organ specific. One possible explanation for the OTU level over-dispersion is competitive exclusion, according to which closely related species cannot co-exist in the same niche^45^. Interestingly, at order level the communities in both fruit and root were different from those in other organs, suggesting that at even at this high a taxonomic level there are taxon specific characteristics that affect how the members of the taxon adapt to the environment.

In studies on actinobacteria, most of the isolates have been identified as members of the genus *Streptomyces*, whereas cultivation independent methods have revealed wider diversity^1–3,46^. Likewise, most of our isolates were identified as *Streptomyces* spp., whereas the most abundant OTU in the *M. toosendan* actinobacterial community was assigned to the slowly growing *Rhodococcus* genera. The most diverse genus in the community was *Tomitella*, members of which were not isolated. The diversity of *Tomitella*, a genus with two known species^47,48^, accentuates that applying novel methods is expected to reveal more and more diversity. To study the actinobacteria further in isolation, the knowledge on community composition may help to develop the cultivation methods.

Actinobacteria isolated from various environments have shown antimicrobial potential^49–51^ *Melia toosendan* hosted potential biocontrol strains that inhibited the growth of a wide range of indicator organisms. As in earlier studies^52,53^, the differences in antimicrobial activity among closely related *Streptomyces* isolates showed that antimicrobial activity is a strain specific characteristic. Isolating and screening all the strains to find the antimicrobially active ones is an enormous task^8^. Thus, cultivation independent methods that could find the most potential ones are called for. Even though many of the genes related to antimicrobial activity are known, predicting the antimicrobial activity based on the presence of those genes has not been successful^52^. Likewise, in this study the genes and their different combinations could not be linked to the growth inhibition potential of the isolates.

We tested the applicability of co-occurrence analysis in detecting antimicrobial activity as an alternative cultivation independent approach, with the rationale that growth inhibiting activity is detectable as negative correlations. To our disappointment, only three OTUs and their corresponding isolate groups were available for comparing co-occurrences and antimicrobial activity. *Streptomyces* OTU4 correlated negatively with a high number of OTUs, and the isolates corresponding to *Streptomyces* OTU4 had high antimicrobial activity. However, for the other two OTUs and their corresponding isolate groups there was no clear relation between the numbers of negative correlations and antimicrobial activity. Thus, to prove the applicability of the co-occurrence analysis in detecting antimicrobially active actinobacteria needs further analyses with more OTUs and their corresponding isolates.

In summary, most of the isolates from *Melia toosendan* tree were identified as *Streptomyces* spp., whereas based on amplicon sequencing the most abundant OTU was assigned to *Rhodococcus*, and *Tomitella* was the most diverse genus. Most of the isolates showed antimicrobial activity. The idea that antimicrobial activity can be detected using a cultivation independent approach and co-occurrence analysis must be further tested in controlled experiments.

## Electronic supplementary material

Supplementary Tables and Figures
