# Supplementary material for: Actinobacteria associated with Chinaberry tree are diverse and show antimicrobial activity
Source: Sci Rep. 2018 Jul 23;8:11103. doi: 10.1038/s41598-018-29442-2 (PMC6056502; doi:10.1038/s41598-018-29442-2)
Supplement: Supplementary file 1 — Supplementary Tables and Figures [file 41598_2018_29442_MOESM1_ESM.docx]

**Actinobacteria associated with Chinaberry tree are diverse and show antimicrobial activity**

Ke Zhao^1a^*, Jing Li^1a^, Meiling Shen^1^, Qiang Chen^1^, Maoke Liu^2^, Xiaolin Ao^1^, Decong Liao^1^, Yunfu Gu^1^, Kaiwei Xu^1^, Menggen Ma^1^, Xiumei Yu^1^, Quanju Xiang^1^, Ji Chen^1^, Xiaoping Zhang^1^, Petri Penttinen^3,4^*

^1^ Department of Microbiology, College of Resource and Environmental Sciences, Sichuan Agricultural University, Yaan 625000, P. R. China

^2^ Biotechnology Center, Rice and Sorghum Research Institute, Sichuan Academy of Agricultural Sciences, Luzhou 646100, P. R. China

^3^ Zhejiang Provincial Key Laboratory of Carbon Cycling in Forest Ecosystems and Carbon Sequestration, School of Environmental & Resource Sciences, Zhejiang Agriculture & Forestry University, Linan 311300, P. R. China

^4^ Ecosystems and Environment Research Programme, University of Helsinki, Helsinki, Fin-00014, Finland

**Supplementary information**

Table S1. The locations of sites where *Melia toosendan* bark, fruit, leaf, root and stem samples were collected.

| Sampling site | Longitude (E) | Latitude (N) |
| --- | --- | --- |
| Suining | 105°38′09.21″ | 30°26′15.00″ |
| Ya’an | 102°59′25.39″ | 29°58′45.05″ |
| Ziyang | 105°26′10.23″ | 30°00′38.19″ |
| Mianyang | 105°06′25.64″ | 30°58′23.99″ |
| Xichang | 102°19′17.59″ | 27°41′51.15″ |
| Jiangtang | 104°26′45.40″ | 30°47′49.27″ |
| Xindu | 104°02′39.34″ | 30°47′31.54″ |

Table S2. Diversity of actinobacterial communities in *Melia toosendan* tree. The average numbers of unique operational taxonomic units (OTUs) and sequences detected by 16S rRNA amplicon sequencing, and the inverse Simpson diversity index in different organs.

| Organ | Number of  OTUs | Number of Sequences | Inverse Simpson |
| --- | --- | --- | --- |
| Bark | 325 ± 260* | 183545±101703 | 4.2 ± 3.9 |
| Fruit | 187± 73 | 186858± 109000 | 2.2 ± 1.3 |
| Leaf | 177 ± 96 | 143381± 41554 | 2.5 ± 2.1 |
| Root | 298± 124 | 110577± 42971 | 2.2 ± 1.2 |
| Stem | 189± 33 | 184169± 114633 | 1.8 ± 0.7 |
| * Average ± standard deviation (n=7) | | |  |

Table S3. Actinobacteria isolated from *Melia toosendan*, and the biosynthetic genes carried by the isolates.

| **Isolate** | **Sampling site** | **Organ** | **Most similar strain (similarity)** | **Biosynthetic gene^a^** | | | |
| --- | --- | --- | --- | --- | --- | --- | --- |
|  |  |  |  | **Halo** | **PKSI** | **PKSII** | **NRPS** |
| SCAU8244 | Xichang | Bark | *Actinokineospora mzabensis* DSM 45961^T^ (KJ504177) 99.26% | − | * | − | − |
| SCAU8245 | Xindou | Bark | *Actinomadura geliboluensis* A8036^T^ (HQ157187) 100% | − | − | − | − |
| SCAU8238 | Xichang | Bark | *Amycolatopsis pretoriensis* NRRL B-24133^T^ (AY183356) 98.62% | − | − | * | − |
| SCAU8248 | Suining | Bark | *Arthrobacter chlorophenolicus* CP001341 100% | − | − | − | − |
| SCAU8241 | Jingtang | Fruit | *Brevibacterium epidermidis* ATCC 35514^T^ (X76565) 98.79% | − | − | − | − |
| SCAU8208 | Xindou | Bark | *Cellulosimicrobium funkei* ATCC BAA-886^T^ (AY501364) 100% | − | − | − | − |
| SCAU8116 | Jingtang | Fruit | *Cellulosimicrobium funkei* ATCC BAA-886^T^ (AY501364) 100% | − | − | − | − |
| SCAU8246 | Ya'an | Bark | *Gordonia westfalica* DSM 44215^T^ (AJ312907) 99.64% | − | − | − | − |
| SCAU8237 | Xichang | Root | *Kitasatospora paranensis* DSM 44788^T^ (AY442268) 99.19% | − | − | − | − |
| SCAU8240 | Xindou | Root | *Kocuria rosea* DSM 20447^T^ (X87756) 99.54% | − | − | − | − |
| SCAU8243 | Ziyang | Bark | *Leucobacter tardus* DSM 19811^T^ (AM940158) 99.85% | − | − | − | − |
| SCAU8109 | Mianyang | Root | *Micromonospora chaiyaphumensis* JCM 12873^T^ (AB196710) 98.72% | − | − | − | − |
| SCAU8155 | Xichang | Stem | *Micromonospora citrea* DSM 43903^T^ (X92617) 98.4% | * | − | * | − |
| SCAU8111 | Ziyang | Stem | *Micromonospora citrea* DSM 43903^T^ (X92617) 98.53% | − | − | * | − |
| SCAU8164 | Suining | Root | *Micromonospora coriariae* DSM 44875^T^ (AJ784008) 99% | − | − | * | − |
| SCAU8151 | Ziyang | Bark | *Micromonospora costi* NBRC 109518^T^ (AB981048) 98.12% | − | − | − | − |
| SCAU8170 | Ziyang | Stem | *Micromonospora peucetia* DSM 43363^T^ (X92603) 99.1% | − | − | − | − |
| SCAU8143 | Mianyang | Root | *Micromonospora peucetia* DSM 43363^T^ (X92603) 99.7% | − | * | * | − |
| SCAU8162 | Ya'an | Root | *Micromonospora peucetia* DSM 43363^T^ (X92603) 99.76%) | − | * | * | − |
| SCAU8141 | Ziyang | Stem | *Micromonospora saelicesensis* DSM 44871^T^ (AJ783993) 94.25% | − | * | − | − |
| SCAU8110 | Ya'an | Root | *Micromonospora saelicesensis* DSM 44871^T^ (AJ783993) 99.11% | * | − | * | − |
| SCAU8107 | Xindou | Bark | *Micromonospora schwarzwaldensis* HKI0641T (KC517406) 99.77% | − | − | − | − |
| SCAU8139 | Ziyang | Root | *Micromonospora schwarzwaldensis* HKI0641^T^ (KC517406) 99.79% | − | − | − | − |
| SCAU8136 | Ziyang | Bark | *Micromonospora schwarzwaldensis* HKI0641^T^ (KC517406) 99.79% | − | − | − | − |
| SCAU8153 | Jingtang | Bark | *Micromonospora schwarzwaldensis* HKI0641^T^ (KC517406) 99.8% | − | * | − | − |
| SCAU8146 | Xichang | Root | *Micromonospora schwarzwaldensis* HKI0641^T^ (KC517406) 99.79% | − | * | − | − |
| SCAU8154 | Suining | Root | *Micromonospora schwarzwaldensis* HKI0641^T^ (KC517406) 98.6% | * | − | − | − |
| SCAU8147 | Mianyang | Stem | *Micromonospora schwarzwaldensis* HKI0641^T^ (KC517406) 99.79% | * | * | * | − |
| SCAU8144 | Ya'an | Bark | *Micromonospora schwarzwaldensis* HKI0641^T^ (KC517406) 99.78% | − | * | − | − |
| SCAU8250 | Ya'an | Root | *Micromonospora schwarzwaldensis* HKI0641^T^ (KC517406) 99.79% | * | − | * | − |
| SCAU8251 | Mianyang | Bark | *Micromonospora siamensis* TT2-4^T^ (AB193565) 98.78% | − | * | − | − |
| SCAU8226 | Suining | Root | *Micromonospora siamensis* TT2-4^T^ (AB193565) 98.75% | − | − | − | − |
| SCAU8165 | Jingtang | Leaf | *Nocardiopsis alba* DSM 43377^T^ (X97883) 100% | − | − | * | − |
| SCAU8105 | Xindou | Leaf | *Nocardiopsis alba* DSM 43377^T^ (X97883) 100% | − | − | * | − |
| SCAU8123 | Mianyang | Root | *Pseudonocardia carboxydivorans* JCM 14827^T^ (EF114314) 99.78% | − | − | − | − |
| SCAU8149 | Ziyang | Root | *Pseudonocardia cypriaca* KT2142^T^ (HQ157191) 96.89% | − | − | − | − |
| SCAU8104 | Jingtang | Leaf | *Rhodococcus enclensis* DSM 45688^T^ (HQ858009) 99.79% | − | − | − | * |
| SCAU8231 | Suining | Stem | *Streptomyces alboflavus* NRRL B-2373^T^ (AB184775) 99.8% | − | − | − | − |
| SCAU8214 | Xindou | Bark | *Streptomyces albogriseolus* NRRL B-1305^T^ (AJ494865) 99.17% | − | − | − | * |
| SCAU8185 | Suining | Stem | *Streptomyces albogriseolus* NRRL B-1305^T^ (AJ494865) 99.38% | − | − | − | − |
| SCAU8122 | Suining | Root | *Streptomyces albogriseolus* NRRL B-1305^T^ (AJ494865) 99.39% | − | − | * | − |
| SCAU8124 | Jingtang | Bark | *Streptomyces albogriseolus* NRRL B-1305^T^ (AJ494865) 99.58% | − | − | * | − |
| SCAU8228 | Jingtang | Root | *Streptomyces albogriseolus* NRRL B-1305^T^ (AJ494865) 99.45% | − | − | − | − |
| SCAU8223 | Ziyang | Root | *Streptomyces alboniger* NBRC 12738^T^ (AB184111) 99.79% | − | − | * | − |
| SCAU8127 | Xindou | Leaf | *Streptomyces alboniger* NBRC 12738^T^ (AB184111) 99.79% | * | − | * | − |
| SCAU8171 | Jingtang | Bark | *Streptomyces althioticus* NRRL B-3981^T^ (AY999791) 98.68% | − | − | − | − |
| SCAU8156 | Jingtang | Root | *Streptomyces althioticus* NRRL B-3981^T^ (AY999791) 98.38% | − | − | * | − |
| SCAU8166 | Ya'an | Stem | *Streptomyces althioticus* NRRL B-3981^T^ (AY999791) 98.76% | − | − | * | − |
| SCAU8112 | Jingtang | Fruit | *Streptomyces althioticus* NRRL B-3981^T^ (AY999791) 98.7% | − | − | − | − |
| SCAU8132 | Suining | Root | *Streptomyces ambofaciens* ATCC 23877^T^ (M27245) 99.8% | − | − | * | − |
| SCAU8140 | Xichang | Fruit | *Streptomyces ambofaciens* ATCC 23877^T^ (M27245) 99.79% | − | − | * | − |
| SCAU8190 | Suining | Fruit | *Streptomyces ambofaciens* ATCC 23877^T^ (M27245) 99.79% | − | * | * | − |
| SCAU8209 | Jingtang | Stem | *Streptomyces ambofaciens* ATCC 23877^T^ (M27245) 99.79% | − | − | * | − |
| SCAU8205 | Suining | Leaf | *Streptomyces ambofaciens* ATCC 23877^T^ (M27245) 99.79% | − | − | * | − |
| SCAU8194 | Xindou | Bark | *Streptomyces ambofaciens* ATCC 23877^T^ (M27245) 99.79% | − | − | − | − |
| SCAU8213 | Mianyang | Fruit | *Streptomyces ambofaciens* ATCC 23877^T^ (M27245) 99.79% | * | − | − | − |
| SCAU8158 | Xindou | Stem | *Streptomyces ambofaciens* ATCC 23877^T^ (M27245) 99.8% | − | * | − | − |
| SCAU8234 | Jingtang | Leaf | *Streptomyces anulatus* NRRL B-2000^T^ (DQ026637) 100% | − | − | − | − |
| SCAU8235 | Ziyang | Root | *Streptomyces anulatus* NRRL B-2000^T^ (DQ026637) 100% | − | − | * | − |
| SCAU8222 | Xindou | Stem | *Streptomyces anulatus* NRRL B-2000^T^ (DQ026637) 100% | − | * | * | − |
| SCAU8137 | Xindou | Leaf | *Streptomyces anulatus* NRRL B-2000^T^ (DQ026637) 100% | − | − | * | * |
| SCAU8148 | Jingtang | Bark | *Streptomyces anulatus* NRRL B-2000^T^ (DQ026637) 100% | − | − | * | − |
| SCAU8218 | Ziyang | Bark | *Streptomyces anulatus* NRRL B-2000T (DQ026637) 99.38% | − | − | * | − |
| SCAU8119 | Ziyang | Leaf | *Streptomyces anulatus* NRRL B-2000^T^ (DQ026637) 100% | − | − | * | * |
| SCAU8252 | Xichang | Stem | *Streptomyces badius* NRRL B-2567^T^ (AY999783) 100% | − | − | − | − |
| SCAU8210 | Jingtang | Leaf | *Streptomyces badius* NRRL B-2567^T^ (AY999783) 99.17% | − | − | − | − |
| SCAU8200 | Ya'an | Bark | *Streptomyces cinerochromogenes* NBRC 13822^T^ (AB184507) 98.75% | * | − | * | − |
| SCAU8102 | Jingtang | Bark | *Streptomyces coelescens* DSM 40421^T^ (AF503496) 100% | − | − | * | * |
| SCAU8189 | Suining | Root | *Streptomyces coelescens* DSM 40421^T^ (AF503496) 100% | − | − | * | − |
| SCAU8225 | Jingtang | Stem | *Streptomyces coelescens* DSM 40421^T^ (AF503496) 100% | * | * | − | − |
| SCAU8128 | Jingtang | Leaf | *Streptomyces coelescens* DSM 40421^T^ (AF503496) 100% | − | − | * | * |
| SCAU8131 | Xichang | Fruit | *Streptomyces coelescens* DSM 40421^T^ (AF503496) 100% | − | − | − | − |
| SCAU8169 | Mianyang | Root | *Streptomyces costaricanus* NBRC 100773^T^ (AB249939) 100% | * | − | − | − |
| SCAU8216 | Suining | Bark | *Streptomyces costaricanus* NBRC 100773^T^ (AB249939) 99.62% | − | * | * | − |
| SCAU8183 | Jingtang | Root | *Streptomyces cyaneus* NRRL B-2296^T^ (AF346475) 99.38% | − | − | − | − |
| SCAU8125 | Ziyang | Root | *Streptomyces ederensis* NBRC 15410^T^ (AB184658) 99.58% | − | − | * | − |
| SCAU8232 | Xichang | Bark | *Streptomyces ederensis* NBRC 15410^T^ (AB184658) 99.59% | * | − | * | − |
| SCAU8193 | Xindou | Leaf | *Streptomyces enissocaesilis* NRRL B-16365^T^ (DQ026641) 100% | − | * | * | * |
| SCAU8195 | Xichang | Root | *Streptomyces enissocaesilis* NRRL B-16365^T^ (DQ026641) 100% | − | * | * | * |
| SCAU8176 | Xindou | Bark | *Streptomyces enissocaesilis* NRRL B-16365^T^ (DQ026641) 100% | − | * | * | − |
| SCAU8177 | Jingtang | Leaf | *Streptomyces enissocaesilis* NRRL B-16365^T^ (DQ026641) 100% | − | * | * | − |
| SCAU8191 | Jingtang | Root | *Streptomyces enissocaesilis* NRRL B-16365^T^ (DQ026641) 100% | − | * | * | − |
| SCAU8175 | Jingtang | Stem | *Streptomyces enissocaesilis* NRRL B-16365^T^ (DQ026641) 100% | − | − | * | − |
| SCAU8187 | Suining | Bark | *Streptomyces enissocaesilis* NRRL B-16365^T^ (DQ026641) 100% | − | * | * | − |
| SCAU8184 | Suining | Root | *Streptomyces enissocaesilis* NRRL B-16365^T^ (DQ026641) 100% | − | * | * | − |
| SCAU8196 | Mianyang | Leaf | *Streptomyces enissocaesilis* NRRL B-16365^T^ (DQ026641) 100% | − | − | * | − |
| SCAU8188 | Suining | Leaf | *Streptomyces enissocaesilis* NRRL B-16365^T^ (DQ026641) 100% | − | * | * | − |
| SCAU8174 | Suining | Fruit | *Streptomyces enissocaesilis* NRRL B-16365^T^ (DQ026641) 100% | − | − | * | − |
| SCAU8221 | Ya'an | Fruit | *Streptomyces enissocaesilis* NRRL B-16365^T^ (DQ026641) 100% | − | − | − | * |
| SCAU8211 | Jingtang | Bark | *Streptomyces enissocaesilis* NRRL B-16365^T^ (DQ026641) 100% | − | * | * | − |
| SCAU8202 | Suining | Stem | *Streptomyces enissocaesilis* NRRL B-16365^T^ (DQ026641) 100% | − | − | * | − |
| SCAU8215 | Suining | Root | *Streptomyces enissocaesilis* NRRL B-16365^T^ (DQ026641) 100% | − | − | * | − |
| SCAU8224 | Ya'an | Bark | *Streptomyces enissocaesilis* NRRL B-16365^T^ (DQ026641) 100% | − | − | * | − |
| SCAU8320 | Mianyang | Stem | *Streptomyces enissocaesilis* NRRL B-16365^T^ (DQ026641) 100% | − | * | * | − |
| SCAU8198 | Ya'an | Stem | *Streptomyces enissocaesilis* NRRL B-16365^T^ (DQ026641) 100% | − | − | * | * |
| SCAU8199 | Suining | Stem | *Streptomyces enissocaesilis* NRRL B-16365^T^ (DQ026641) 100% | − | − | * | * |
| SCAU8160 | Xichang | Bark | *Streptomyces exfoliatus* DSM 40060^T^ (AB184324) 100% | − | * | − | * |
| SCAU8113 | Ziyang | Root | *Streptomyces filipinensis* NBRC 12860^T^ (AB184198) 98.54% | − | − | − | − |
| SCAU8134 | Jingtang | Bark | *Streptomyces flavovirens* NBRC 12860^T^ (AB184198) 100% | − | − | * | * |
| SCAU8135 | Ya'an | Fruit | *Streptomyces flavovirens* NBRC 3716^T^ (AB184834) 100% | − | − | * | * |
| SCAU8186 | Suining | Root | *Streptomyces fulvissimus* NBRC 3716^T^ (AB184834) 100% | − | − | * | − |
| SCAU8178 | Mianyang | Fruit | *Streptomyces geysiriensis* NBRC 15413^T^ (AB184661) 100% | − | − | * | * |
| SCAU8192 | Xindou | Stem | *Streptomyces geysiriensis* NBRC 15413^T^ (AB184661) 100% | − | − | * | − |
| SCAU8206 | Mianyang | Root | *Streptomyces geysiriensis* NBRC 15413^T^ (AB184661) 100% | − | − | * | − |
| SCAU8118 | Suining | Bark | *Streptomyces humiferus* DSM 43030^T^ (AF503491) 97.67% | − | − | * | − |
| SCAU8203 | Ya'an | Leaf | *Streptomyces hydrogenans* NBRC 13475^T^ (AB184868) 100% | * | − | * | − |
| SCAU8207 | Ziyang | Root | *Streptomyces hydrogenans* NBRC 13475^T^ (AB184868) 100% | − | − | − | − |
| SCAU8108 | Suining | Stem | *Streptomyces hydrogenans*  NBRC 13475^T^ (AB184868) 100% | − | * | − | − |
| SCAU8201 | Xichang | Bark | *Streptomyces hydrogenans* NBRC 13475^T^ (AB184868) 98.96% | − | − | * | − |
| SCAU8159 | Xindou | Bark | *Streptomyces hydrogenans* NBRC 13475^T^ (AB184868) 100% | − | * | − | − |
| SCAU8161 | Jingtang | Root | *Streptomyces hydrogenans* NBRC 13475^T^ (AB184868) 100% | − | * | − | − |
| SCAU8157 | Xichang | Root | *Streptomyces hydrogenans* NBRC 13475^T^ (AB184868) 100% | − | − | * | * |
| SCAU8182 | Jingtang | Stem | *Streptomyces hydrogenans* NBRC 13475^T^ (AB184868) 100% | * | − | * | − |
| SCAU8180 | Ziyang | Bark | *Streptomyces hydrogenans* NBRC 13475^T^ (AB184868) 100% | * | − | * | − |
| SCAU8129 | Ya'an | Leaf | *Streptomyces hydrogenans* NBRC 13475^T^ (AB184868) 100% | − | * | − | * |
| SCAU8130 | Mianyang | Fruit | *Streptomyces hydrogenans* NBRC 13475^T^ (AB184868) 100% | − | * | − | − |
| SCAU8103 | Ziyang | Root | *Streptomyces kunmingensis* NBRC 14463^T^ (AB184597) 99.15% | * | − | * | − |
| SCAU8204 | Ziyang | Root | *Streptomyces lavendulae subsp. lavendulae* ATCC 19777^T^ (D85116)98.54% | − | − | * | − |
| SCAU8230 | Ya'an | Leaf | *Streptomyces mobaraensis* ATCC 29032^T^ (DQ442528) 98.16% | − | - | * | − |
| SCAU8120 | Mianyang | Root | *Streptomyces nitrosporeus* NBRC 3362^T^ (AB184751) 98.78% | − | − | − | − |
| SCAU8114 | Ziyang | Root | *Streptomyces nogalater* JCM 4799^T^ (AB045886) 98.75% | − | − | * | − |
| SCAU8179 | Ziyang | Root | *Streptomyces roseolus* NBRC 12816^T^ (AB184168) 99.38% | − | − | − | − |
| SCAU8117 | Xindou | Root | *Streptomyces roseolus* NBRC 12816^T^ (AB184168) 100% | − | * | * | * |
| SCAU8133 | Ziyang | Bark | *Streptomyces roseolus* NBRC 12816^T^ (AB184168) 99.32% | − | − | * | − |
| SCAU8229 | Ziyang | Root | *Streptomyces shaanxiensis*  CCNWHQ 0031^T^ (FJ465151) 98.57% | * | − | * | − |
| SCAU8167 | Suining | Root | *Streptomyces tendae* ATCC 19812^T^ (D63873) 99.78% | − | − | * | * |
| SCAU8101 | Xindou | Stem | *Streptomyces tendae* ATCC 19812^T^ (D63873) 99.97% | − | * | * | * |
| SCAU8212 | Jingtang | Leaf | *Streptomyces violarus* NBRC 13104^T^ (AB184316) 99.58% | − | − | * | − |
| SCAU8181 | Ziyang | Root | *Streptomyces violarus* NBRC 13104^T^ (AB184316) 99.58% | * | − | * | − |
| SCAU8217 | Ziyang | Root | *Streptomyces viridochromogenes* ATCC 14920^T^ (DQ442555) 99.58% | − | − | − | * |
| SCAU8115 | Jingtang | Root | *Streptosporangium amethystogenes subsp. amethystogenes* ATCC 33327^T^ (X89935) 100% | * | − | − | − |
| SCAU8106 | Xichang | Root | *Streptosporangium amethystogenes subsp. amethystogenes* ATCC 33327T (X89935) 100% | * | − | * | − |
| SCAU8152 | Ziyang | Root | *Streptosporangium amethystogenes subsp. amethystogenes* ATCC 33327T (X89935) 100% | * | − | − | − |
| SCAU8142 | Ziyang | Root | *Streptosporangium amethystogenes subsp. amethystogenes* ATCC 33327T (X89935) 100% | * | − | − | − |
| SCAU8163 | Xindou | Bark | *Tsukamurella strandjordii* ATCC BAA-173^T^ (AF283283) 99.3% | − | * | * | − |

**^a^** KS domain of PKSI, the KS domain of PKSII, the adenylation domain of NRPS and FADH_2_-dependent halogenase gene of halogenation pathway.

**^b^** 1) *Colletotrichum orbiculare* (SAUM 5312), 2) *Fusarium oxysporum* (SAUM 5429), 3) *Altemaria solani* (SAUM 5230), 4) *Magnaporthe grisea* (SAUM 5411), 5) *Curvularia lunata* (SAUM 5429), 6) *Gibberella saubinetii* (SAUM 5456), 7) *Bacillus subtilis* (SAUM 5139), 8) *Staphylococcus aureus* (ATCC25923) and 9) *Escherichia coli* (ATCC 35218).

Table S4. Number of OTUs and abundances of actinobacterial taxa detected as endophytes of *Melia toosendan* tree by 16S rRNA gene amplicon sequencing.

| **Taxon** | **Number of OTUs** | **Abundance** |
| --- | --- | --- |
| **Class** |  |  |
| Acidimicrobiia | 161 | 4186 |
| Actinobacteria | 7416 | 5659824 |
| **Order** |  |  |
| Acidimicrobiales | 161 | 4186 |
| Actinomycetales | 35 | 816 |
| Bifidobacteriales | 2 | 8 |
| Catenulisporales | 11 | 58 |
| Corynebacteriales | 2062 | 4054930 |
| Frankiales | 925 | 234427 |
| Glycomycetales | 6 | 724 |
| Kineosporiales | 95 | 7955 |
| Micrococcales | 2152 | 887645 |
| Micromonosporales | 167 | 10137 |
| Propionibacteriales | 1244 | 82979 |
| Pseudonocardiales | 393 | 42829 |
| Streptomycetales | 284 | 337005 |
| Streptosporangiales | 37 | 311 |
| **Family** |  |  |
| Acidimicrobiaceae | 40 | 2408 |
| Acidimicrobiales_Incertae_Sedis | 39 | 446 |
| Acidothermaceae | 155 | 61229 |
| Actinomycetaceae | 35 | 816 |
| Actinospicaceae | 8 | 48 |
| Beutenbergiaceae | 69 | 1098 |
| Bifidobacteriaceae | 2 | 8 |
| Bogoriellaceae | 23 | 1714 |
| Brevibacteriaceae | 31 | 1885 |
| Catenulisporaceae | 3 | 10 |
| Cellulomonadaceae | 486 | 146905 |
| Corynebacteriaceae | 144 | 49476 |
| Corynebacteriales_Incertae_Sedis | 1000 | 202089 |
| Cryptosporangiaceae | 64 | 1964 |
| Demequinaceae | 12 | 321 |
| Dermabacteraceae | 23 | 2444 |
| Dermacoccaceae | 45 | 273 |
| Dermatophilaceae | 90 | 2135 |
| Dietziaceae | 30 | 23831 |
| Frankiaceae | 310 | 59614 |
| Geodermatophilaceae | 269 | 80467 |
| Glycomycetaceae | 6 | 724 |
| Iamiaceae | 31 | 1332 |
| Intrasporangiaceae | 223 | 11181 |
| Jonesiaceae | 5 | 11 |
| Kineosporiaceae | 95 | 7955 |
| Microbacteriaceae | 733 | 377302 |
| Micrococcaceae | 139 | 67521 |
| Micrococcales_Incertae_Sedis | 57 | 3607 |
| Micromonosporaceae | 167 | 10137 |
| Mycobacteriaceae | 259 | 1492800 |
| Nakamurellaceae | 80 | 21485 |
| Nocardiaceae | 619 | 2282232 |
| Nocardioidaceae | 839 | 44688 |
| Nocardiopsaceae | 6 | 159 |
| Promicromonosporaceae | 33 | 2364 |
| Propionibacteriaceae | 405 | 38291 |
| Pseudonocardiaceae | 393 | 42829 |
| Rarobacteraceae | 4 | 13 |
| Ruaniaceae | 73 | 947 |
| Sanguibacteraceae | 106 | 267924 |
| Segniliparaceae | 1 | 1 |
| Sporichthyaceae | 47 | 9668 |
| Streptomycetaceae | 284 | 337005 |
| Streptosporangiaceae | 19 | 81 |
| Thermomonosporaceae | 12 | 71 |
| Tsukamurellaceae | 9 | 4501 |
| **Genus** |  |  |
| Acaricomes | 9 | 15 |
| Aciditerrimonas | 39 | 446 |
| Acidothermus | 155 | 61229 |
| Acrocarpaspora | 2 | 17 |
| Actinoalloteichus | 12 | 1176 |
| Actinobaculum | 2 | 9 |
| Actinocorallia | 2 | 12 |
| Actinokineospora | 2 | 26 |
| Actinomadura | 6 | 50 |
| Actinomyces | 13 | 763 |
| Actinomycetospora | 29 | 12511 |
| Actinophytocola | 29 | 5734 |
| Actinoplanes | 23 | 4832 |
| Actinospica | 8 | 48 |
| Actinosynnema | 1 | 20 |
| Actinotalea | 48 | 1761 |
| Aeromicrobium | 368 | 6168 |
| Aestuariimicrobium | 1 | 1 |
| Agreia | 18 | 52 |
| Agrococcus | 10 | 5084 |
| Agromyces | 15 | 1585 |
| Alpinimonas | 16 | 32270 |
| Amnibacterium | 31 | 24489 |
| Amycolatopsis | 12 | 4080 |
| Angustibacter | 34 | 3152 |
| Aquipuribacter | 7 | 12 |
| Arsenicicoccus | 29 | 87 |
| Arthrobacter | 53 | 4950 |
| Asanoa | 1 | 4 |
| Beutenbergia | 8 | 517 |
| Bifidobacterium | 2 | 8 |
| Blastococcus | 154 | 70162 |
| Brachybacterium | 13 | 2433 |
| Brevibacterium | 31 | 1885 |
| Candidatus_Planktoluna | 2 | 4 |
| Candidatus_Rhodoluna | 31 | 119 |
| Catellatospora | 6 | 68 |
| Catenulispora | 3 | 10 |
| Catenuloplanes | 11 | 112 |
| Cellulomonas | 10 | 60358 |
| Cellulosimicrobium | 6 | 1105 |
| Citricoccus | 18 | 58899 |
| CL500-29_marine_group | 4 | 27 |
| Clavibacter | 4 | 16 |
| Corynebacterium | 144 | 49476 |
| Couchioplanes | 6 | 26 |
| Crossiella | 25 | 45 |
| Cryocola | 5 | 25 |
| Cryptosporangium | 29 | 1677 |
| Curtobacterium | 43 | 31220 |
| Dactylosporangium | 4 | 194 |
| Demequina | 1 | 32 |
| Demetria | 19 | 62 |
| Dermabacter | 3 | 3 |
| Dermatophilus | 5 | 18 |
| Devriesea | 1 | 1 |
| Diaminobutyricimonas | 13 | 16 |
| Dietzia | 30 | 23831 |
| Flexivirga | 4 | 69 |
| Fodinicola | 35 | 287 |
| Frankia | 38 | 7893 |
| Friedmanniella | 64 | 7608 |
| Frigoribacterium | 55 | 87539 |
| Geodermatophilus | 67 | 3939 |
| Georgenia | 6 | 11 |
| Glaciibacter | 19 | 28 |
| Glycomyces | 6 | 724 |
| Goodfellowiella | 17 | 22 |
| Gordonia | 35 | 24015 |
| Helcobacillus | 6 | 7 |
| Herbiconiux | 59 | 2074 |
| hgcI_clade | 2 | 54 |
| Homoserinimonas | 52 | 208 |
| Hoyosella | 95 | 176004 |
| Iamia | 31 | 1332 |
| Ilumatobacter | 36 | 2381 |
| Intrasporangium | 70 | 356 |
| Isoptericola | 8 | 133 |
| Janibacter | 14 | 732 |
| Jatrophihabitans | 272 | 51721 |
| Jiangella | 88 | 1654 |
| Jonesia | 5 | 11 |
| Kibdelosporangium | 24 | 2840 |
| Kineococcus | 5 | 1710 |
| Kineosphaera | 9 | 64 |
| Kineosporia | 10 | 360 |
| Kitasatospora | 12 | 15 |
| Knoellia | 12 | 122 |
| Kocuria | 24 | 1221 |
| Kribbella | 37 | 4587 |
| Kribbia | 9 | 15 |
| Kutzneria | 8 | 9 |
| Kytococcus | 3 | 19 |
| Lechevalieria | 1 | 1 |
| Leifsonia | 30 | 85 |
| Lentzea | 1 | 14 |
| Leucobacter | 3 | 78 |
| Longispora | 19 | 51 |
| Luteimicrobium | 57 | 3607 |
| Luteipulveratus | 18 | 123 |
| Luteococcus | 21 | 94 |
| Lysinimicrobium | 11 | 289 |
| Lysinimonas | 8 | 473 |
| Marisediminicola | 10 | 11 |
| Marmoricola | 137 | 3173 |
| Microbacterium | 30 | 176273 |
| Microbispora | 2 | 14 |
| Microcella | 6 | 84 |
| Micrococcus | 7 | 1999 |
| Microlunatus | 70 | 1241 |
| Micromonospora | 4 | 3958 |
| Micropruina | 42 | 4492 |
| Millisia | 230 | 784 |
| Miniimonas | 7 | 81 |
| Mobilicoccus | 2 | 2 |
| Mobiluncus | 1 | 3 |
| Modestobacter | 43 | 6366 |
| MWH-Ta3 | 3 | 16 |
| Myceligenerans | 4 | 4 |
| Mycetocola | 45 | 539 |
| Mycobacterium | 259 | 1492800 |
| Nakamurella | 80 | 21485 |
| Nesterenkonia | 5 | 232 |
| Nocardia | 12 | 1656 |
| Nocardioides | 267 | 30319 |
| Nocardiopsis | 3 | 148 |
| Nonomuraea | 2 | 4 |
| Oceanitalea | 17 | 1703 |
| Oerskovia | 127 | 67487 |
| Okibacterium | 26 | 33 |
| Ornithinicoccus | 11 | 20 |
| Paraoerskovia | 155 | 16715 |
| Phycicoccus | 38 | 5653 |
| Phytohabitans | 6 | 114 |
| Pilimelia | 6 | 48 |
| Piscicoccus | 73 | 2051 |
| Planobispora | 1 | 1 |
| Planomonospora | 1 | 1 |
| Planosporangium | 7 | 8 |
| Plantactinospora | 20 | 44 |
| Plantibacter | 21 | 93 |
| Polymorphospora | 7 | 46 |
| Prauserella | 21 | 62 |
| Promicromonospora | 15 | 1122 |
| Propionibacterium | 30 | 19462 |
| Propioniciclava | 15 | 55 |
| Propioniferax | 30 | 88 |
| Pseudoclavibacter | 47 | 287 |
| Pseudonocardia | 142 | 10263 |
| Quadrisphaera | 46 | 2733 |
| Rarobacter | 4 | 13 |
| Rathayibacter | 19 | 84 |
| Rhodococcus | 139 | 2252543 |
| Rhodoglobus | 4 | 24 |
| Rothia | 13 | 177 |
| Ruania | 73 | 947 |
| Rugosimonospora | 2 | 2 |
| Saccharomonospora | 3 | 37 |
| Saccharopolyspora | 42 | 874 |
| Salinispora | 2 | 2 |
| Sanguibacter | 106 | 267924 |
| Schumannella | 69 | 14486 |
| Sediminihabitans | 140 | 584 |
| Segniliparus | 1 | 1 |
| Serinibacter | 54 | 500 |
| Serinicoccus | 9 | 46 |
| Sinomonas | 6 | 22 |
| Sinosporangium | 8 | 27 |
| Skermania | 143 | 2406 |
| Smaragdicoccus | 44 | 780 |
| Sphaerisporangium | 2 | 12 |
| Spirilliplanes | 11 | 36 |
| Spirillospora | 4 | 9 |
| Sporichthya | 45 | 9614 |
| Stackebrandtia | 6 | 15 |
| Streptacidiphilus | 77 | 224 |
| Streptomyces | 195 | 336766 |
| Streptosporangium | 1 | 5 |
| SV1-8 | 2 | 2 |
| Terrabacter | 7 | 28 |
| Terracoccus | 3 | 37 |
| Tessaracoccus | 32 | 3596 |
| Tetrasphaera | 10 | 4073 |
| Thermasporomyces | 27 | 441 |
| Thermobifida | 3 | 11 |
| Tomitella | 899 | 26085 |
| Trueperella | 19 | 41 |
| Tsukamurella | 9 | 4501 |
| Umezawaea | 18 | 20 |
| Verrucosispora | 17 | 194 |
| Williamsia | 6 | 48 |
| Virgisporangium | 7 | 383 |
| Yaniella | 3 | 6 |
| Yonghaparkia | 5 | 5 |

Table S5 Antimicrobial activity of actinobacteria isolated from *Melia toosendan* based on widths of inhibition zones (mm; average ± standard deviation, n = 3) against nine indicator organisms.

| **Isolate** | **Indicator organism** | |  |  |  |  |  |  |  |
| --- | --- | --- | --- | --- | --- | --- | --- | --- | --- |
|  | **1** | **2** | **3** | **4** | **5** | **6** | **7** | **8** | **9** |
| *Actinokineospora mzabensis* SCAU8244 | 0 | 5.9±0.46 | 0 | 13.7±0.45 | 13.9±0.53 | 0 | 0 | 12.4±0.42 | 0 |
| *Actinomadura geliboluensis* SCAU8245 | 0 | 0 | 0 | 0 | 0 | 0 | 0 | 0 | 0 |
| *Amycolatopsis pretoriensis* SCAU8238 | 0 | 0 | 15.2±0.40 | 14.3±0.60 | 18.0±0.37 | 0 | 0 | 11.1±.0.06 | 6.4±0.12 |
| *Arthrobacter chlorophenolicus* SCAU8248 | 0 | 12.4±0.70 | 0 | 0 | 0 | 14.5±0.35 | 0 | 0 | 0 |
| *Brevibacterium epidermidis* SCAU8241 | 0 | 0 | 8.8±0.44 | 0 | 0 | 0 | 0 | 0 | 0 |
| *Cellulosimicrobium funkei* SCAU8208 | 0 | 0 | 0 | 0 | 0 | 0 | 0 | 0 | 0 |
| *Cellulosimicrobium funkei* SCAU8116 | 0 | 0 | 0 | 0 | 0 | 0 | 0 | 5.9±0.45 | 0 |
| *Gordonia westfalica* SCAU8246 | 0 | 0 | 0 | 0 | 0 | 0 | 0 | 0 | 0 |
| *Kitasatospora paranensis* SCAU8237 | 0 | 15.0±0.17 | 0 | 0 | 0 | 0 | 0 | 0 | 7.7±0.29 |
| *Kocuria rosea* SCAU8240 | 0 | 0 | 0 | 0 | 0 | 0 | 0 | 0 | 0 |
| *Leucobacter tardus* SCAU8243 | 0 | 0 | 0 | 0 | 0 | 0 | 0 | 0 | 0 |
| *Micromonospora chaiyaphumensis* SCAU8109 | 12.8±0.76 | 7.3±1.15 | 18.9±0.57 | 11.1±0.50 | 17.2±0.21 | 12.3±0.15 | 0 | 6.0±0.40 | 0 |
| *Micromonospora citrea* SCAU8155 | 0 | 0 | 0 | 0 | 0 | 0 | 13.2±0.25 | 0 | 7.5±0.50 |
| *Micromonospora citrea* SCAU8111 | 0 | 0 | 0 | 0 | 0 | 0 | 0 | 0 | 0 |
| *Micromonospora coriariae* SCAU8164 | 0 | 0 | 0 | 0 | 0 | 5.7±0.25 | 0 | 0 | 8.0±0.06 |
| *Micromonospora costi* SCAU8151 | 0 | 0 | 0 | 0 | 0 | 0 | 0 | 0 | 0 |
| *Micromonospora peucetia* SCAU8170 | 0 | 6.2±0.80 | 19.7±0.65 | 5.8±0.32 | 15.4±0.37 | 16.9±0.06 | 0 | 13.8±0.26 | 7.3±0.29 |
| *Micromonospora peucetia* SCAU8143 | 11.9±0.84 | 6.8±1.04 | 15.2±0.56 | 0 | 0 | 0 | 0 | 7.1±0.20 | 8.4±0.34 |
| *Micromonospora peucetia* SCAU8162 |  | 0 | 0 | 0 | 0 | 0 | 0 | 0 | 0 |
| *Micromonospora saelicesensis* SCAU8141 | 17.9±0.36 | 8.4±0.57 | 17.7±0.61 | 5.5±0.35 | 14.0±0.25 | 0 | 0 | 6.1±0.12 | 8.1±0.29 |
| *Micromonospora saelicesensis* SCAU8110 | 13.2±0.46 | 0 | 12.7±1.15 | 0 | 16.4±0.50 | 15.1±0.23 | 0 | 0 | 0 |
| *Micromonospora schwarzwaldensis* SCAU8107 | 0 | 0 | 0 | 0 | 0 | 0 | 0 | 10.3±0.26 | 10.6±0.36 |
| *Micromonospora schwarzwaldensis* SCAU8139 | 0 | 8.6±0.69 | 0 | 0 | 14.7±0.37 | 0 | 0 | 0 | 0 |
| *Micromonospora schwarzwaldensis* SCAU8136 | 0 | 0 | 0 | 0 | 12.9±0.34 | 13.2±0.44 | 0 | 0 | 0 |
| *Micromonospora schwarzwaldensis* SCAU8153 | 0 | 0 | 5.7±0.66 | 0 | 0 | 0 | 0 | 0 | 0 |
| *Micromonospora schwarzwaldensis* SCAU8146 | 5.7±0.45 | 3.8±0.17 | 8.0±0.40 | 0 | 18.2±0.46 | 0 | 0 | 0 | 0 |
| *Micromonospora schwarzwaldensis* SCAU8154 | 0 | 0 | 13.2±0.67 | 0 | 0 | 0 | 0 | 0 | 0 |
| *Micromonospora schwarzwaldensis* SCAU8147 | 12.8±0.42 | 0 | 14.8±0.29 | 0 | 19.8±0.33 | 15.3±0.52 | 0 | 0 | 0 |
| *Micromonospora schwarzwaldensis* SCAU8144 | 0 | 0 | 0 | 0 | 0 | 0 | 0 | 0 | 0 |
| *Micromonospora schwarzwaldensis* SCAU8250 | 18.7±0.55 | 0 | 20.3±0.67 | 7.7±0.60 | 20.7±0.25 | 0 | 0 | 10.7±0.21 | 13.4±0.32 |
| *Micromonospora siamensis* SCAU8251 | 12.7±0.72 | 0 | 0 | 7.2±0.30 | 12.2±0.17 | 0 | 5.6±0.35 | 0 | 0 |
| *Micromonospora siamensis* SCAU8226 | 0 | 0 | 12.7±0.65 | 13.9±0.35 | 15.3±0.45 | 0 | 0 | 14.2±0.06 | 6.5±0.26 |
| *Nocardiopsis alba* SCAU8165 | 0 | 0 | 0 | 0 | 0 | 0 | 0 | 0 | 0 |
| *Nocardiopsis alba* SCAU8105 | 0 | 0 | 0 | 0 | 0 | 0 | 0 | 0 | 0 |
| *Pseudonocardia carboxydivorans* SCAU8123 | 15.1±0.60 | 6.6±0.81 | 16.8±0.60 | 8.8±0.40 | 16.1±0.29 | 16.5±0.21 | 0 | 12.5±0.15 | 14.5±0.32 |
| *Pseudonocardia cypriaca* SCAU8149 | 16.7±0.30 | 0 | 18.5±0.32 | 6.7±0.40 | 18.1±0.52 | 19.4±0.53 | 0 | 15.1±0.70 | 5.8±0.17 |
| *Rhodococcus enclensis* SCAU8104 | 13.7±0.69 | 0 | 0 | 14.1±0.21 | 0 | 15.4±0.40 | 0 | 12.4±0.46 | 5.5±0.46 |
| *Streptomyces alboflavus* SCAU8231 | 19.7±0.72 | 8.8±0.21 | 17.3±0.56 | 14.8±0.30 | 5.4±0.29 | 16.8±0.30 | 0 | 14.6±0.45 | 8.5±0.23 |
| *Streptomyces albogriseolus* SCAU8214 | 0 | 0 | 0 | 0 | 0 | 0 | 0 | 13.4±0.32 | 5.1±0.17 |
| *Streptomyces albogriseolus* SCAU8185 | 0 | 0 | 0 | 0 | 0 | 0 | 11.6±0.10 | 6.3±0.21 | 0 |
| *Streptomyces albogriseolus* SCAU8122 | 0 | 0 | 0 | 0 | 0 | 0 | 10.1±0.12 | 6.5±0.20 | 0 |
| *Streptomyces albogriseolus* SCAU8124 | 0 | 0 | 0 | 0 | 0 | 0 | 15.2±0.35 | 0 | 0 |
| *Streptomyces albogriseolus* SCAU8228 | 0 | 0 | 0 | 0 | 0 | 0 | 0 | 0 | 0 |
| *Streptomyces alboniger* SCAU8223 | 0 | 0 | 0 | 6.9±0.45 | 6.5±0.21 | 6.5±0.26 | 0 | 0 | 0 |
| *Streptomyces alboniger* SCAU8127 | 0 | 0 | 0 | 7.0±0.15 | 0 | 7.5±0.40 | 0 | 0 | 0 |
| *Streptomyces althioticus* SCAU8171 | 5.7±0.32 | 14.1±1.08 | 0 | 9.0±0.56 | 14.2±0.29 | 13.3±0.21 | 5.4±0.29 | 7.1±0.35 | 0 |
| *Streptomyces althioticus* SCAU8156 | 17.6±0.61 | 12.2±0.70 | 19.1±0.38 | 5.4±0.41 | 17.6±0.21 | 18.2±0.31 | 0 | 16.4±0.17 | 0 |
| *Streptomyces althioticus* SCAU8166 | 0 | 0 | 0 | 0 | 0 | 0 | 0 | 0 | 0 |
| *Streptomyces althioticus* SCAU8112 | 18.5±0.40 | 0 | 15.8±0.82 | 13.2±0.45 | 17.2±0.28 | 17.4±0.40 | 5.6±0.23 | 12.4±**0.06** | 5.7±0.17 |
| *Streptomyces ambofaciens* SCAU8132 | 0 | 0 | 0 | 0 | 0 | 0 | 0 | 0 | 0 |
| *Streptomyces ambofaciens* SCAU8140 | 0 | 0 | 0 | 0 | 0 | 0 | 0 | 6.0±0.12 | 0 |
| *Streptomyces ambofaciens* SCAU8190 | 0 | 0 | 0 | 0 | 0 | 0 | 0 | 0 | 13.5±0.15 |
| *Streptomyces ambofaciens* SCAU8209 | 6.0±0.59 | 0 | 0 | 0 | 5.3±0.25 | 0 | 0 | 5.6±0.17 | 7.1±0.38 |
| *Streptomyces ambofaciens* SCAU8205 | 0 | 0 | 0 | 0 | 0 | 0 | 0 | 5.9±0.15 | 11.5±0.10 |
| *Streptomyces ambofaciens* SCAU8194 | 0 | 0 | 0 | 0 | 0 | 0 | 0 | 8.2±0.05 | 12.4±0.12 |
| *Streptomyces ambofaciens* SCAU8213 | 16±0.46 | 8.8±0.70 | 17.6±0.31 | 13.9±0.43 | 5.9±0.16 | 16.4±0.15 | 0 | 11.3±0.29 | 14.3±0.29 |
| *Streptomyces ambofaciens* SCAU8158 | 0 | 0 | 0 | 0 | 0 | 0 | 0 | 8.1±0.23 | 0 |
| *Streptomyces anulatus* SCAU8234 | 0 | 0 | 0 | 0 | 0 | 0 | 0 | 0 | 0 |
| *Streptomyces anulatus* SCAU8235 | 0 | 0 | 0 | 0 | 5.3±0.21 | 0 | 0 | 0 | 0 |
| *Streptomyces anulatus* SCAU8222 | 0 | 0 | 0 | 7.2±0.32 | 0 | 0 | 0 | 0 | 0 |
| *Streptomyces anulatus* SCAU8137 | 14.9±0.45 | 0 | 0 | 8.5±0.35 | 13.3±0.29 | 0 | 0 | 0 | 0 |
| *Streptomyces anulatus* SCAU8148 | 0 | 0 | 0 | 0 | 6.4±0.23 | 0 | 0 | 0 | 0 |
| *Streptomyces anulatus* SCAU8218 | 19.2±0.55 | 0 | 0 | 19.±0.66 | 7.5±0.29 | 0 | 0 | 0 | 0 |
| *Streptomyces anulatus* SCAU8119 | 0 | 0 | 18.8±0.67 | 0 | 13.0±0.39 | 6.0±0.06 | 0 | 8.7±0.23 | 14.5±0.61 |
| *Streptomyces badius* SCAU8252 | 0 | 0 | 0 | 7.1±0.41 | 0 | 0 | 0 | 0 | 0 |
| *Streptomyces badius* SCAU8210 | 18.2±0.76 | 0 | 0 | 0 | 6.5±0.22 | 0 | 0 | 0 | 0 |
| *Streptomyces cinerochromogenes* SCAU8200 | 0 | 7.2±1.11 | 0 | 15.3±0.40 | 14.5±0.33 | 16.5±0.26 | 6.9±0.10 | 0 | 10.4±0.18 |
| *Streptomyces coelescens* SCAU8102 | 0 | 0 | 0 | 0 | 0 | 0 | 0 | 6.0±0.12 | 0 |
| *Streptomyces coelescens* SCAU8189 | 0 | 0 | 0 | 7.3±0.36 | 6.6±0.05 | 0 | 0 | 5.20±0.35 | 0 |
| *Streptomyces coelescens* SCAU8225 | 0 | 0 | 0 | 13.9±0.35 | 0 | 0 | 6.1±0.23 | 19.2±0.40 | 5.9±0.18 |
| *Streptomyces coelescens* SCAU8128 | 0 | 0 | 0 | 0 | 0 | 0 | 0 | 0 | 0 |
| *Streptomyces coelescens* SCAU8131 | 0 | 0 | 0 | 0 | 13.8±0.29 | 0 | 0 | 0 | 0 |
| *Streptomyces costaricanus* SCAU8169 | 0 | 5.9±0.10 | 0 | 7.6±0.30 | 13.3±0.17 | 12.9±0.21 | 0 | 0 | 0 |
| *Streptomyces costaricanus* SCAU8216 | 19.8±0.96 | 18.1±0.55 | 16.5±0.30 | 19.±0.55 | 19.1±0.34 | 15.4±0.26 | 13.4±0.21 | 18.2±0.29 | 11.9±0.49 |
| *Streptomyces cyaneus* SCAU8183 | 14.9±0.87 | 0 | 0 | 0 | 7.3±0.14 | 0 | 0 | 0 | 0 |
| *Streptomyces ederensis* SCAU8125 | 0 | 0 | 0 | 0 | 0 | 0 | 0 | 0 | 0 |
| *Streptomyces ederensis* SCAU8232 | 0 | 0 | 13.8±0.25 | 0 | 0 | 5.5±0.31 | 0 | 12.2±0.35 | 14.5±0.38 |
| *Streptomyces enissocaesilis* SCAU8193 | 17.2±0.76 | 0 | 6.8±0.55 | 7.4±0.45 | 6.5±0.29 | 13.1±0.35 | 6.3±0.06 | 18.3±0.26 | 12.8±0.34 |
| *Streptomyces enissocaesilis* SCAU8195 | 18.5±0.55 | 14.5±0.78 | 0 | 5.1±0.23 | 19.4±0.45 | 14.4±0.20 | 0 | 0 | 0 |
| *Streptomyces enissocaesilis* SCAU8176 | 16.4±0.57 | 5.67±0.29 | 6.4±0.31 | 6.3±0.40 | 17.2±0.31 | 14.9±0.23 | 7.6±0.33 | 18.1±0.29 | 15.8±0.22 |
| *Streptomyces enissocaesilis* SCAU8177 | 12.1±0.15 | 7.8±0.47 | 0 | 8.4±0.49 | 12.7±0.25 | 16.1±0.23 | 6.3±0.12 | 0 | 16.3±0.39 |
| *Streptomyces enissocaesilis* SCAU8191 | 12.1±0.78 | 5.8±0.25 | 0 | 5.5±0.47 | 5.4±0.24 | 9.6±0.36 | 0 | 15.1±0.23 | 17.7±0.34 |
| *Streptomyces enissocaesilis* SCAU8175 | 14.2±1.23 | 12.6±0.40 | 0 | 15.2±0.55 | 5.8±0.29 | 0 | 6.6±0.31 | 15.5±0.46 | 15.7±0.12 |
| *Streptomyces enissocaesilis* SCAU8187 | 12.6±0.81 | 8.2±0.73 | 0 | 8.2±0.32 | 5.3±0.38 | 7.1±0.15 | 12.3±0.39 | 16.2±0.35 | 19.0±0.28 |
| *Streptomyces enissocaesilis* SCAU8184 | 14.3±1.21 | 7.1±0.79 | 0 | 5.4±0.26 | 7.2±0.25 | 5.4±0.26 | 12.9±0.17 | 16.4±0.21 | 16.5±0.40 |
| *Streptomyces enissocaesilis* SCAU8196 | 7.4±0.71 | 5.5±0.43 | 13.0±0.56 | 11.9±0.36 | 6.4±0.33 | 11.3±0.31 | 0 | 13.2±0.46 | 6.6±0.29 |
| *Streptomyces enissocaesilis* SCAU8188 | 11.7±0.42 | 14.6±0.45 | 0 | 7.4±0.40 | 0 | 5.23±0.32 | 0 | 15.1±0.17 | 16.8±0.42 |
| *Streptomyces enissocaesilis* SCAU8174 | 11.3±0.35 | 0 | 8.2±0.40 | 0 | 11.5±0.37 | 6.1±0.17 | 5.3±0.23 | 16.2±0.21 | 17.5±0.38 |
| *Streptomyces enissocaesilis* SCAU8221 | 15.1±0.44 | 0 | 0 | 14.7±0.26 | 7.5±0.33 | 5.2±0.20 | 6.1±0.23 | 16.2±0.17 | 17.7±0.28 |
| *Streptomyces enissocaesilis* SCAU8211 | 9.3±0.64 | 6.2±0.95 | 13.6±0.35 | 8.1±0.25 | 6.7±0.17 | 5.4±0.29 | 0 | 15.8±0.06 | 15.6±0.16 |
| *Streptomyces enissocaesilis* SCAU8202 | 15.0±0.60 | 6.0±0.89 | 13.0±0.36 | 19.±0.50 | 18.1±0.34 | 17.2±0.26 | 6.2±0.06 | 11.3±0.29 | 16.7±0.47 |
| *Streptomyces enissocaesilis* SCAU8215 | 7.9±0.17 | 6.2±0.20 | 0 | 14.5±0.66 | 9.0±0.29 | 18.4±0.45 | 0 | 12.4±0.15 | 16.1±0.56 |
| *Streptomyces enissocaesilis* SCAU8224 | 13.1±0.78 | 13.0±0.15 | 15.1±0.62 | 5.6±0.25 | 11.0±0.45 | 0 | 5.1±0.06 | 20.0±0.23 | 18.3±0.44 |
| *Streptomyces enissocaesilis* SCAU8320 | 20.8±0.53 | 0 | 0 | 12.4±0.26 | 15.3±0.36 | 13.4±0.35 | 0 | 20.2±0.40 | 13.5±0.39 |
| *Streptomyces enissocaesilis* SCAU8198 | 19.9±1.05 | 7.03±0.55 | 0 | 11.6±0.23 | 13.2±0.05 | 13.0±0.49 | 0 | 13.3±0.46 | 12.2±0.33 |
| *Streptomyces enissocaesilis* SCAU8199 | 13.6±0.69 | 8.63±0.64 | 12.1±0.21 | 7.4±0.35 | 0 | 10.4±0.15 | 8.6±0.06 | 18.1±0.23 | 19.2±0.43 |
| *Streptomyces exfoliatus* SCAU8160 | 0 | 0 | 0 | 0 | 0 | 0 | 0 | 0 | 0 |
| *Streptomyces filipinensis* SCAU8113 | 0 | 0 | 0 | 0 | 0 | 0 | 0 | 0 | 0 |
| *Streptomyces flavovirens* SCAU8134 | 0 | 0 | 0 | 5.4±0.40 | 0 | 12.8±0.41 | 0 | 0 | 0 |
| *Streptomyces flavovirens* SCAU8135 | 3.1±0.21 | 0 | 0 | 5.2±0.34 | 14.3±0.12 | 16.5±0.15 | 0 | 0 | 0 |
| *Streptomyces fulvissimus* SCAU8186 | 0 | 0 | 0 | 6.3±0.30 | 0 | 0 | 0 | 0 | 20.8±0.59 |
| *Streptomyces geysiriensis* SCAU8178 | 12.8±0.25 | 0 | 5.9±0.36 | 11.9±0.30 | 7.3±0.17 | 12.2±0.32 | 0 | 19.4±0.46 | 19.1±0.60 |
| *Streptomyces geysiriensis* SCAU8192 | 14.2±0.70 | 0 | 8.7±0.31 | 11.7±0.55 | 8.2±0.12 | 19.8±0.35 | 0 | 14.5±0.29 | 12.2±0.28 |
| *Streptomyces geysiriensis* SCAU8206 | 0 | 5.8±0.32 | 0 | 0 | 7.6±0.12 | 6.23±0.31 | 6.2±0.17 | 17.8±0.69 | 10.9±0.31 |
| *Streptomyces humiferus* SCAU8118 | 18.5±0.50 | 19.7±0.38 | 19.2±0.32 | 11.6±0.34 | 12.5±0.29 | 11.5±0.36 | 0 | 13.7±0.35 | 13.5±0.39 |
| *Streptomyces hydrogenans* SCAU8203 | 6.6±0.67 | 6.6±0.45 | 12.1±0.51 | 0 | 14.8±0.21 | 6.3±0.20 | 0 | 0 | 0 |
| *Streptomyces hydrogenans* SCAU8207 | 7.5±0.50 | 5.7±1.15 | 14.2±0.34 | 6.3±0.43 | 13.2±0.24 | 5.1±0.23 | 0 | 12.5±0.17 | 0 |
| *Streptomyces hydrogenans* SCAU8108 | 8.5±0.41 | 7.30±0.36 | 19.2±0.75 | 13.8±0.77 | 0 | 14.3±0.31 | 0 | 11.3±0.40 | 0 |
| *Streptomyces hydrogenans* SCAU8201 | 8.6±0.75 | 0 | 6.3±0.44 | 6.3±0.45 | 12.2±0.26 | 13.4±0.35 | 0 | 0 | 20.5±0.95 |
| *Streptomyces hydrogenans* SCAU8159 | 0 | 0 | 0 | 5.6±0.26 | 6.4±0.22 | 0 | 0 | 0 | 0 |
| *Streptomyces hydrogenans* SCAU8161 | 0 | 6.7±0.21 | 0 | 13.2±0.35 | 13.5±0.05 | 0 | 0 | 10.5±0.29 | 0 |
| *Streptomyces hydrogenans* SCAU8157 | 0 | 0 | 0 | 0 | 0 | 0 | 0 | 0 | 0 |
| *Streptomyces hydrogenans* SCAU8182 | 18.8±1.16 | 11.7±0.50 | 5.3±0.25 | 8.6±0.25 | 0 | 8.5±0.47 | 0 | 7.4±0.12 | 0 |
| *Streptomyces hydrogenans* SCAU8180 | 17.3±0.52 | 17.1±0.31 | 11.9±0.55 | 5.7±0.26 | 5.6±0.29 | 7.2±0.29 | 0 | 7.2±0.15 | 0 |
| *Streptomyces hydrogenans* SCAU8129 | 0 | 0 | 13.1±0.62 | 0 | 12.3±0.05 | 0 | 0 | 0 | 0 |
| *Streptomyces hydrogenans* SCAU8130 | 0 | 0 | 13.4±0.26 | 3.1±0.30 | 15.2±0.09 | 19.4±0.45 | 3.2±0.15 | 8.7±0.17 | 0 |
| *Streptomyces kunmingensis* SCAU8103 | 0 | 0 | 11.3±0.31 | 0 | 0 | 0 | 0 | 0 | 0 |
| *Streptomyces lavendulae* SCAU8204 | 0 | 0 | 0 | 7.9±0.35 | 6.7±0.22 | 5.4±0.40 | 0 | 0 | 0 |
| *Streptomyces mobaraensis* SCAU8230 | 7.1±0.26 | 15.1±0.35 | 0 | 14.3±0.55 | 13.5±0.29 | 6.2±0.10 | 0 | 11.5±0.32 | 14.2±0.41 |
| *Streptomyces nitrosporeus* SCAU8120 | 0 | 0 | 0 | 0 | 0 | 0 | 0 | 0 | 0 |
| *Streptomyces nogalater* SCAU8114 | 0 | 0 | 0 | 0 | 0 | 0 | 0 | 12.6±0.49 | 12.2±0.37 |
| *Streptomyces roseolus* SCAU8179 | 0 | 0 | 0 | 15.3±0.55 | 0 | 13.7±0.25 | 0 | 9.1±0.26 | 0 |
| *Streptomyces roseolus* SCAU8117 | 0 | 0 | 0 | 7.1±0.30 | 0 | 0 | 0 | 0 | 0 |
| *Streptomyces roseolus* SCAU8133 | 0 | 0 | 0 | 0 | 0 | 0 | 0 | 0 | 0 |
| *Streptomyces shaanxiensis* SCAU8229 | 0 | 13.3±0.75 | 0 | 9.2±0.30 | 10.6±0.31 | 6.6±0.26 | 0 | 0 | 0 |
| *Streptomyces tendae* SCAU8167 | 0 | 0 | 0 | 0 | 0 | 0 | 0 | 0 | 0 |
| *Streptomyces tendae* SCAU8101 | 0 | 0 | 0 | 0 | 0 | 0 | 0 | 6.0±0.25 | 0 |
| *Streptomyces violarus* SCAU8212 | 7.5±0.50 | 0 | 0 | 5.3±0.32 | 10.1±0.14 | 7.4±0.21 | 0 | 0 | 0 |
| *Streptomyces violarus* SCAU8181 | 0 | 0 | 0 | 14.1±0.45 | 5.1±0.09 | 0 | 0 | 0 | 0 |
| *Streptomyces viridochromogenes* SCAU8217 | 0 | 0 | 0 | 0 | 5.8±0.28 | 0 | 0 | 0 | 0 |
| *Streptosporangium amethystogenes* SCAU8115 | 13.1±0.76 | 0 | 18.3±0.67 | 8.3±0.25 | 18.1±0.19 | 8.4±0.15 | 0 | 13.1±0.67 | 7.69±0.30 |
| *Streptosporangium amethystogenes* SCAU8106 | 0 | 0 | 0 | 0 | 0 | 0 | 0 | 0 | 5.64±0.47 |
| *Streptosporangium amethystogenes* SCAU8152 | 0 | 0 | 0 | 0 | 0 | 0 | 0 | 0 | 0 |
| *Streptosporangium amethystogenes* SCAU8142 | 0 | 0 | 0 | 0 | 0 | 0 | 0 | 14.4±0.49 | 0 |
| *Tsukamurella strandjordii* SCAU8163 | 13.2±0.35 | 11.9±0.60 | 13.7±0.50 | 12.8±0.37 | 12.3±0.22 | 10.2±0.32 | 0 | 10.5±0.29 | 0 |

**^a^** 1) *Colletotrichum orbiculare* (SAUM 5312), 2) *Fusarium oxysporum* (SAUM 5429), 3) *Altemaria solani* (SAUM 5230), 4) *Magnaporthe grisea* (SAUM 5411), 5) *Curvularia lunata* (SAUM 5429), 6) *Gibberella saubinetii* (SAUM 5456), 7) *Bacillus subtilis* (SAUM 5139), 8) *Staphylococcus aureus* (ATCC25923) and 9) *Escherichia coli* (ATCC 35218).


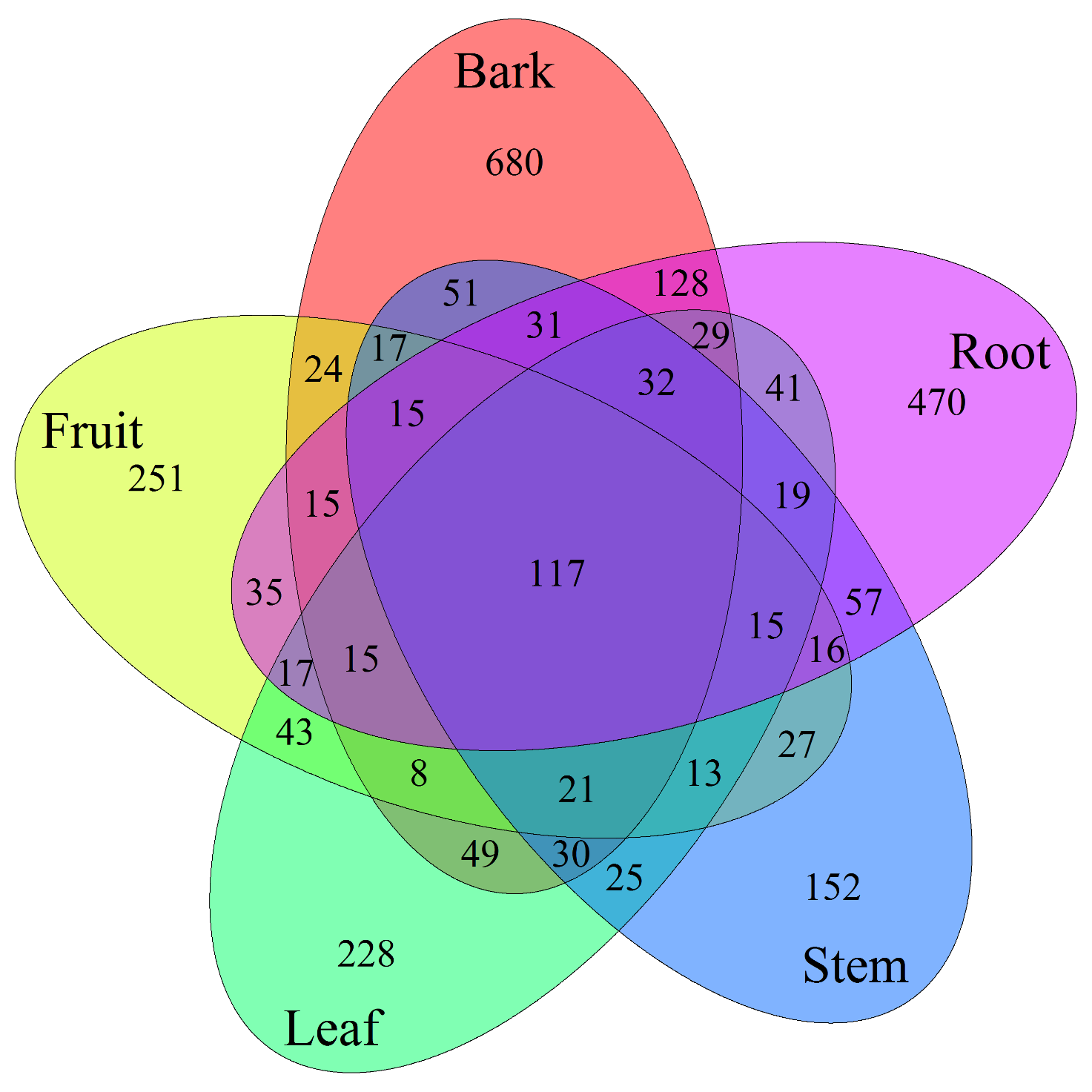


Figure S1. Number of actinobacterial OTUs in different organs of *Melia toosendan* tree, based on 16S rRNA gene amplicon sequencing.

C

D

B

A

Figure S2 The scanning electron micrographs of actinobacteria isolated from *Melia toosendan*. A) SCAU8195, B) SCAU8216, C) SCAU8241, D) SCAU8248.

**A**

**B**

**C**

**D**


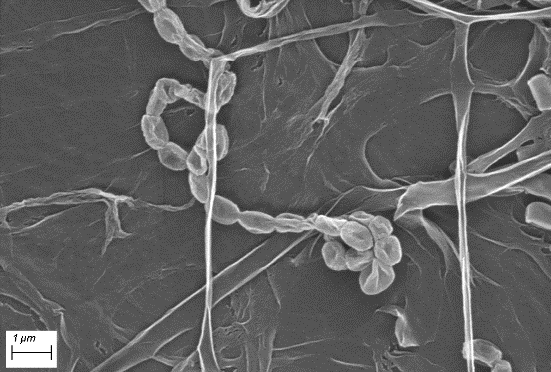

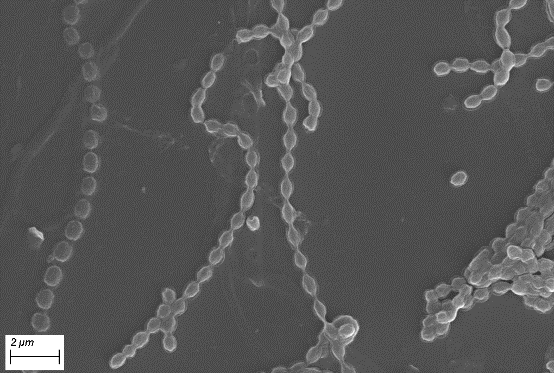

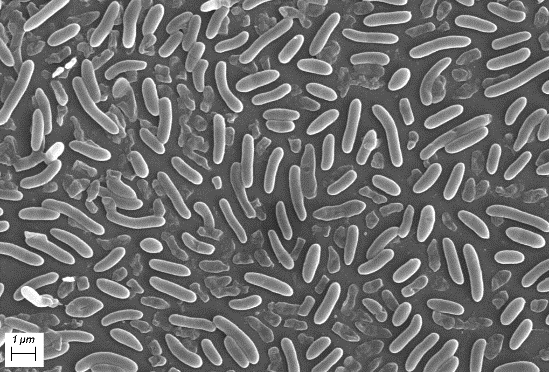

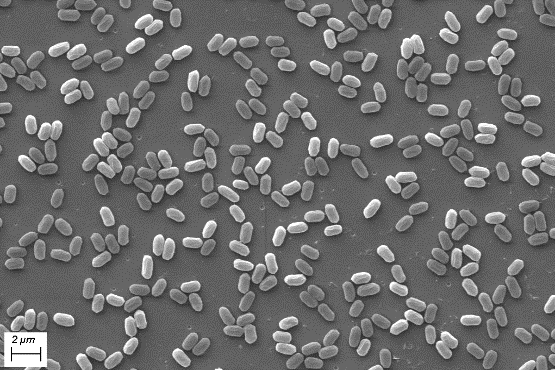


b10 (KY388877)

b27 (KY388894)

b54 (KY388920)

b43 (KY388910)

*Streptomyces* *flavogriseus* (KP096288)

*Streptomyces* *griseus* (NR_115143)

b13 (KY388880)

b46 (KY388913)

*Streptomyces* *microflavus* (KP657976)

b52 (KY388918)

b16 (KY388883)

b6 (KY388873)

b40 (KY388907)

*Streptomyces* sp. (KX777584)

b42 (KY388909)

b8 (KY388875)

b15 (KY388882)

b48 (KY388915)

*Aeromicrobium* *panaciterrae* (NR_041382)

b19 (KY388886)

b31 (KY388898)

*Nocardioides* sp. (KP893899)

*Aeromicrobium* sp. (KJ649724)

b2 (KY388869)

b36 (KY388903)

*Rhodococcus* *maanshanensis* (AF416566)

b47 (KY388914)

*Rhodococcus* *erythropolis* (LK995417)

*Rhodococcus* *qingshengii* (KP980622)

b30 (KY388897)

b11 (KY388878)

b18 (KY388885)

b44 (KY388911)

b41 (KY388908)

b7 (KY388874)

*Corynebacterium* *appendicis* (AJ314919)

b24 (KY388891)

b21 (KY388888)

*Amycolatopsis* *coloradensis* (AJ293753)

*Pseudonocardia* *oroxyli* (DQ343154)

b29 (KY388896)

*Gordonia* *hirsuta* (X93485)

b25 (KY388892)

b39 (KY388906)

b5 (KY388872)

*Mycobacterium* sp. (FN298348)

b28 (KY388895)

*Mycobacterium* *hodleri* (KT950757)

b56 (KY388922)

b38 (KY388905)

b4 (KY388871)

b1 (KY388868)

b34 (KY388901)

*Nakamurella* *panacisegetis* KCTC 19426 (HE599560)

b3 (KY388870)

Uncultured Nakamurellaceae bacterium (HQ462476)

b49 (KY388916)

b9 (KY388876)

Uncultured Micrococcineae bacterium (KX035362)

b55 (KY388921)

b35 (KY388902)

*Blastococcus* sp. (LN62628)

b22 (KY388889)

*Micromonospora* *violae* (KC161209)

b14 (KY388881)

b32 (KY388899)

*Promicromonospora* *sukumoe* (AB023375)

*Brevibacterium* *luteolum* (AJ488509)

b23 (KY388890)

b20 (KY388887)

*Amnibacterium* *kyonggiense* (FJ527819)

*Leifsonia* sp. (KR265652)

b50 (KY388917)

b33 (KY388900)

*Leifsonia* sp. (KP899246)

*Microbacterium* *foliorum* (AJ249780)

b26 (KY388893)

b45 (KY388912)

b17 (KY388884)

b12 (KY388879)

100

100

99

100

100

99

93

100

98

84

75

91

99

68

79

58

73

98

98

76

88

99

72

87

94

82

67

96

74

65

73

56

99

77

52

99

74

96

78

58

50

73

86

63

98

64

52

70

0.02

b53 (KY388919)

Figure S3. Relationships between the 16S rRNA gene sequences of endophytic actinobacteria from *Melia toosendon* detected by DGGE and the closest matching sequences in NCBI GenBank.


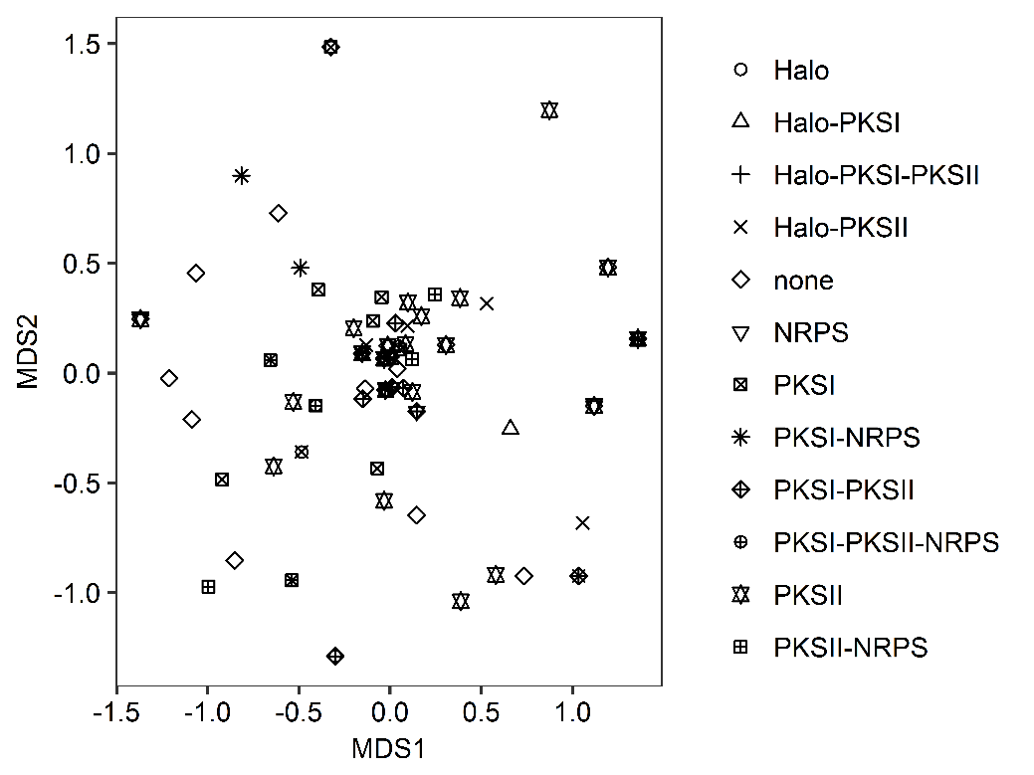


Figure S4. Nonmetric multidimensional scaling based on the Bray–Curtis dissimilarities between isolates from *Melia toosendan* carrying different combinations of antimicrobial biosynthetic genes. Bray–Curtis dissimilarities of the isolates that inhibited the growth of at least one indicator strain were calculated based on the presence/absence of inhibition. PKSI = the KS domain of PKSI, PKSII = the KS domain of PKSII, NRPS = the adenylation domain of NRPS, and Halo = FADH_2_-dependent halogenase gene of halogenation pathway.
